# From plasma membrane to lysosomes: expanding roles of TMEM63/OSCA channels in mechanosensation and intracellular signaling

**DOI:** 10.3389/fphar.2026.1909797

**Published:** 2026-08-07

**Authors:** Serap Erdogmus, Gurkan Mollaoglu, Ursula Storch

**Affiliations:** 1 Clinical Pharmacy, Institute of Pharmacy, University of Regensburg, Regensburg, Germany; 2 Department of Microbiology, Heersink School of Medicine, University of Alabama at Birmingham, Birmingham, AL, United States; 3 Immunology Institute, Heersink School of Medicine, University of Alabama at Birmingham, Birmingham, AL, United States; 4 O’Neal Comprehensive Cancer Center, University of Alabama at Birmingham, Birmingham, AL, United States

**Keywords:** ion channel, lipid scramblase, lysosome, mechanosensation, mechanotransduction, OSCA, TMEM63

## Abstract

Mechanosensation enables cells to detect physical forces, including membrane stretch, pressure, shear stress, and osmotic stress, and to convert them into biochemical and electrical signals that regulate cellular function. Among the emerging families of mechanosensitive proteins, TMEM63/OSCA channels have recently been recognized as evolutionarily conserved mechanically activated ion channels with important roles in cellular and organellar physiology. While mechanosensation has traditionally been studied at the plasma membrane, increasing evidence suggests that intracellular organelles also experience and respond to mechanical cues. Lysosomes are particularly exposed to membrane tension, curvature changes, osmotic fluctuations, and cytoskeletal forces generated during trafficking, fusion-fission dynamics and cargo loading. These observations support an emerging view of lysosomes as dynamic hubs of mechano-responsive signaling in addition to their established degradative functions. Recent studies have identified TMEM63 proteins at both the plasma membrane and lysosomes, where they are proposed to couple mechanical and osmotic stimuli to ion flux, membrane remodeling, and stress-adaptive signaling pathways. In this review, we summarize current advances in the structural biology, gating mechanisms, and physiological functions of TMEM63/OSCA channels with particular emphasis on their emerging roles in lysosomal mechanobiology. We further discuss evidence linking TMEM63/OSCA dysfunction to human channelopathies and highlight key questions regarding mechanosignaling across cellular compartments. By integrating recent findings from mechanobiology, organelle physiology, and disease genetics, this review positions TMEM63 channels as an important molecular link between membrane mechanics, lysosomal function, and cellular homeostasis.

## 1 Introduction

Mechanical forces are fundamental regulators of cellular behavior and tissue homeostasis. Throughout life, cells are continuously exposed to a wide variety of physical stimuli, including membrane stretch, hydrostatic pressure, shear stress, osmotic fluctuations, extracellular matrix stiffness, and forces generated by the surrounding cellular environment. The ability to detect (mechanosensation) and respond (mechanotransduction) to these mechanical cues is essential for numerous physiological processes, including embryonic development, tissue morphogenesis, sensory perception, vascular regulation, respiration, proprioception, immune responses, and cellular migration (Kefauver et al., 2020; Poole, 2022; Douguet and Honore, 2019; Di et al., 2023; Murthy et al., 2017). Conversely, dysregulated mechanosensation and mechanotransduction contribute to the pathogenesis of diverse human diseases, including cardiovascular disorders, pulmonary hypertension, kidney disease, neurodegeneration, chronic inflammation and cancer (Murthy et al., 2017; Wang et al., 2021; Ogino et al., 2024; Otero-Sobrino et al., 2023; Bera et al., 2022; Tang et al., 2023; Lim, 2022; Zeng et al., 2025).

Mechanotransduction refers to the conversion of mechanical stimuli into biochemical and electrical signals that influence cellular function. Although multiple molecular systems contribute to mechanotransduction, mechanosensitive ion channels (MICs) represent one of the most direct and rapid mechanisms by which cells can sense physical forces. By coupling membrane deformation to ion fluxes across biological membranes, these channels enable cells to monitor and respond to changes in their physical environment within milliseconds (Kefauver et al., 2020; Poole, 2022; Douguet and Honore, 2019; Murthy et al., 2017; Becchetti et al., 2019). The importance of mechanosensitive ion channels has become increasingly apparent over the past 2 decades, resulting in the identification of numerous channel families that participate in force sensing throughout evolution (Kefauver et al., 2020; Murthy et al., 2017; Coste et al., 2010; Syeda, 2021; Wu et al., 2017; Arnadottir and Chalfie, 2010).

**FIGURE 1:**
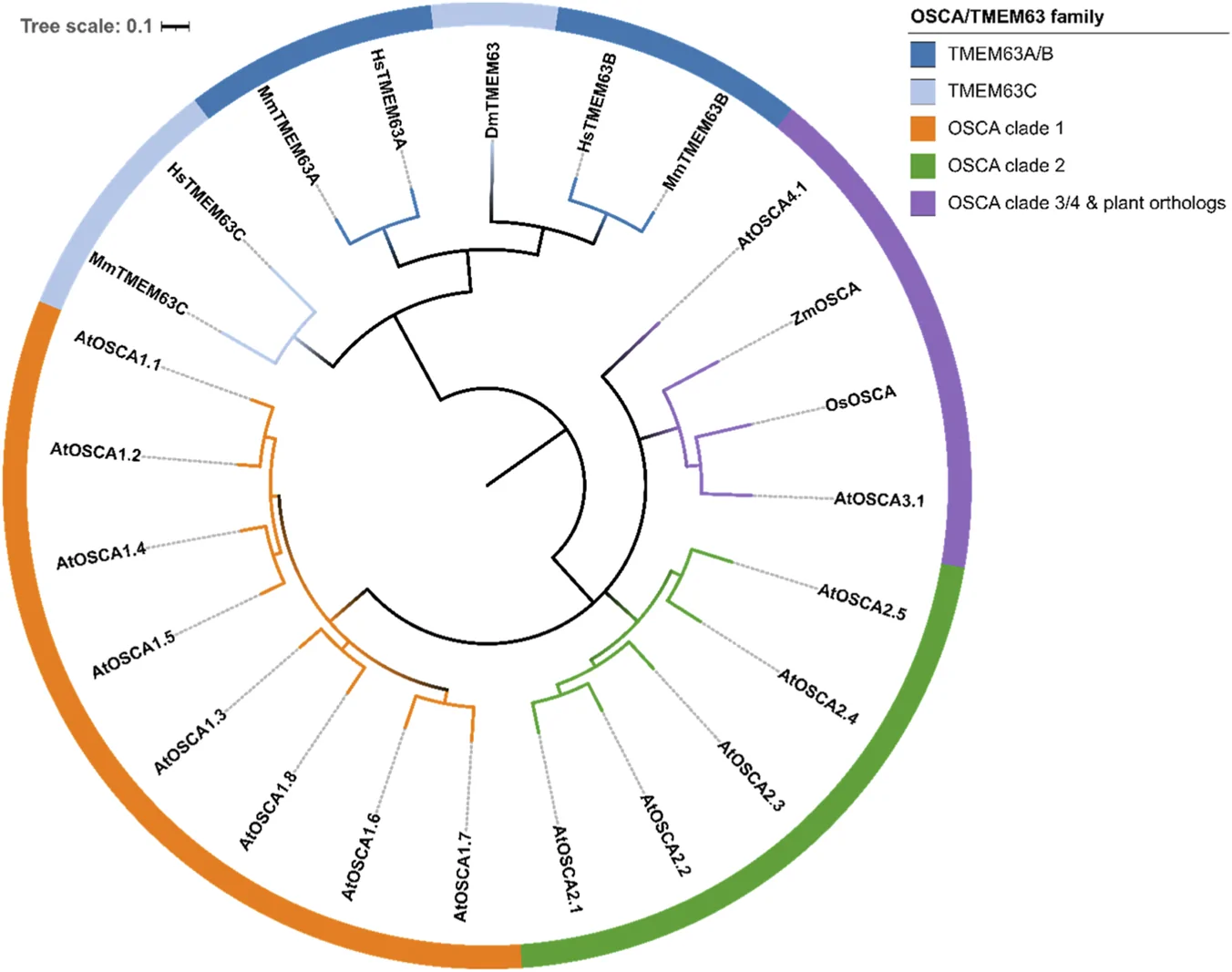
Representative phylogenetic relationships within the evolutionarily conserved OSCA/TMEM63 family. Phylogenetic analysis of selected OSCA/TMEM63 protein sequences from plants and animals, including plant OSCA isoforms from *Arabidopsis thaliana* (At), Oryza sativa (Os), and Zea mays (Zm), together with representative TMEM63 orthologs from *Homo sapiens* (Hs), *Mus musculus* (Mm), and *Drosophila melanogaster* (Dm). The analysis illustrates the broad evolutionary conservation of the OSCA/TMEM63 family across eukaryotes and the separation of animal TMEM63 proteins from major plant OSCA clades. The tree is intended to show representative relationships rather than a complete phylogeny of all OSCA/TMEM63 family members. Branch colors indicate the principal groups shown in the analysis: TMEM63A/B (dark blue), TMEM63C (light blue), OSCA clade 1 (orange), OSCA clade 2 (green), and OSCA clades 3/4 together with representative plant OSCA orthologs from *Arabidopsis thaliana*, Oryza sativa and Zea mays (purple). Branch lengths are proportional to evolutionary distance and reflect sequence divergence among family members. Phylogenetic tree visualization was generated using the Interactive Tree Of Life platform Version 6 (iTOL v6) (Letunic and Bork, 2024).

Among the most recently recognized mechanosensitive channel families are the OSCA/TMEM63 proteins (Murthy et al., 2018). These proteins were initially discovered in plants as hyperosmolarity-responsive calcium-permeable channels that mediate osmotic stress signaling in plants (Hou et al., 2014; Yuan et al., 2014). Subsequent studies revealed that OSCA proteins belong to an evolutionarily conserved family present throughout eukaryotes and mammalian homologs (Figure 1), which are termed TMEM63A, TMEM63B and TMEM63C, and function as mechanically activated ion channels responsive to membrane stretch, osmotic stress, and membrane tension (Murthy et al., 2018; Zhao et al., 2016; Zheng et al., 2023). The evolutionary conservation of these proteins suggests that membrane-based force sensing represents an ancient cellular mechanism that predates the emergence of specialized sensory systems (Figure 1). Recent advances in cryo-electron microscopy (cryo-EM) and structural biology have transformed our understanding of TMEM63 channels. High-resolution structures have revealed unique architectural features that distinguish TMEM63 proteins from other mechanosensitive ion channel families and have provided important insights into their gating mechanisms and interactions with membrane lipids (Zheng et al., 2023; Jojoa-Cruz et al., 2018; Liu et al., 2018; Zhang M. et al., 2018; Maity et al., 2019; Jojoa-Cruz et al., 2024; Zhang M. et al., 2023; Qin et al., 2023; Han et al., 2024; Shan et al., 2024). These studies support the concept that TMEM63 channels operate primarily through direct sensing of membrane mechanics (Teng et al., 2015; Anishkin et al., 2014; Pliotas et al., 2015; Bavi et al., 2023; Martinac et al., 1990; Perozo et al., 2002), placing them among the growing group of inherently mechanosensitive proteins.

**FIGURE 2:**
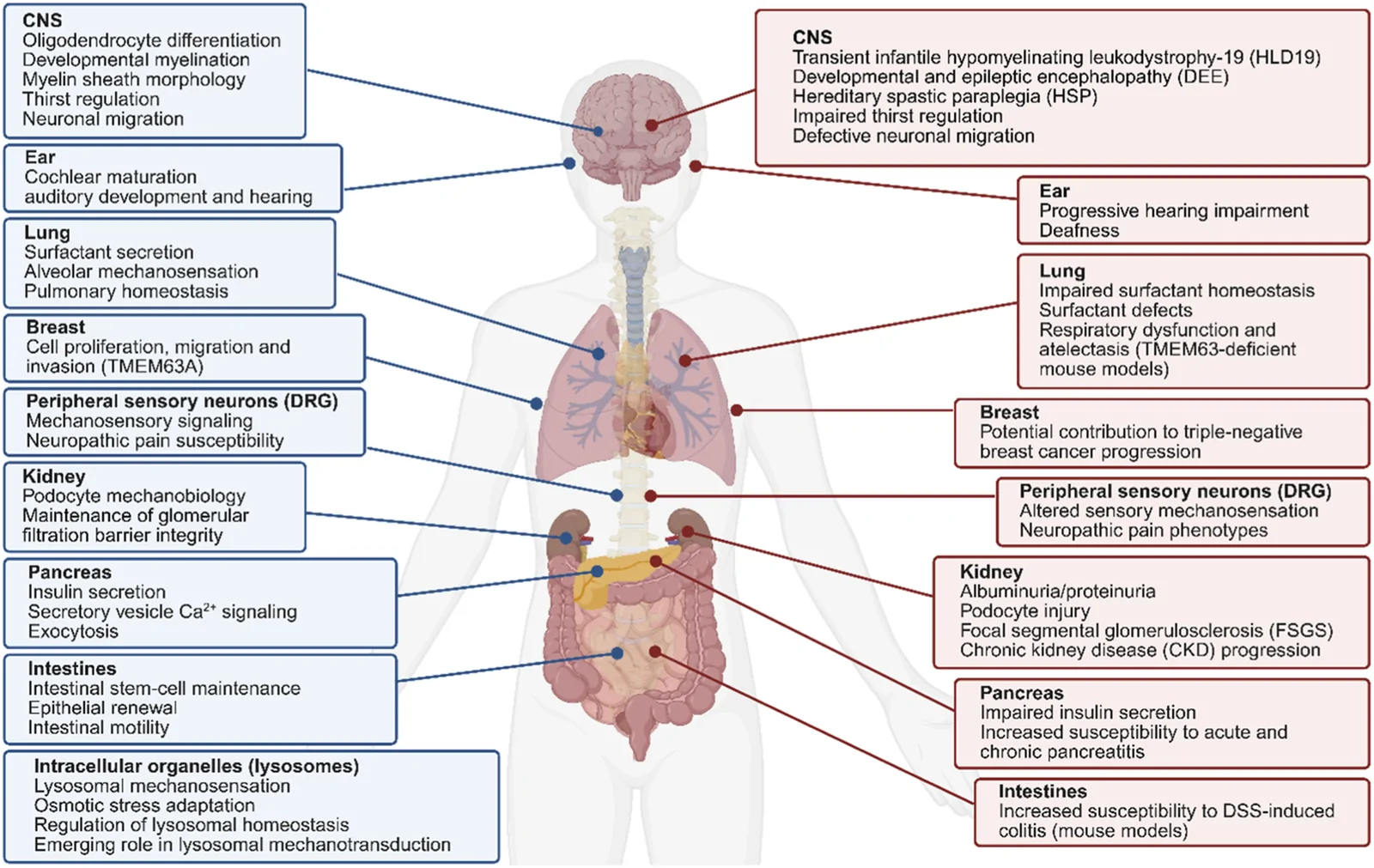
Tissue-specific physiological roles and disease associations of TMEM63 family proteins. Current evidence indicates that TMEM63 proteins contribute to diverse physiological processes across multiple tissues and organ systems, including the central nervous system (CNS), ear, lung, breast, peripheral sensory neurons (DRG), kidney, pancreas, intestines, and lysosomes. Disease associations reflect a combination of human genetic evidence and phenotypes observed in experimental animal models. Created with BioRender.com.

Importantly, the biological functions of TMEM63 proteins extend beyond conventional plasma membrane mechanosensation. Recent evidence demonstrates that TMEM63 family members participate in diverse physiological processes (Figure 2), including oligodendrocyte differentiation and myelination (Wang Y. Y. et al., 2025; Halford et al., 2025; Dereddi et al., 2026), hearing (Du et al., 2020; Ye et al., 2024), thirst regulation (Yang et al., 2024; Zou et al., 2025), pulmonary surfactant secretion (Chen et al., 2024; Hook, 2024), endocrine signaling (Ye et al., 2024), pancreatic (Tsvilovskyy et al., 2024; Patel and Yule, 2024; Tu et al., 2025) and intestinal physiology (Tu et al., 2024), pain sensation (Pu et al., 2023) and renal homeostasis (Orphal et al., 2020; Eisenreich et al., 2020). Furthermore, pathogenic variants in TMEM63 genes have been linked to a growing spectrum of human diseases (Figure 2) (Zheng et al., 2025), including hypomyelinating leukodystrophy (Yoneno et al., 2024; Yan et al., 2019; Tonduti et al., 2021; Fukumura et al., 2022), developmental and epileptic encephalopathy (Sasaki et al., 2024; Vetro et al., 2023), hereditary spastic paraplegia (Tábara et al., 2022), hearing impairment, kidney disorders (Schulz et al., 2019), and cancer (Zhang T. M. et al., 2023) (Figure 2).

**FIGURE 3:**
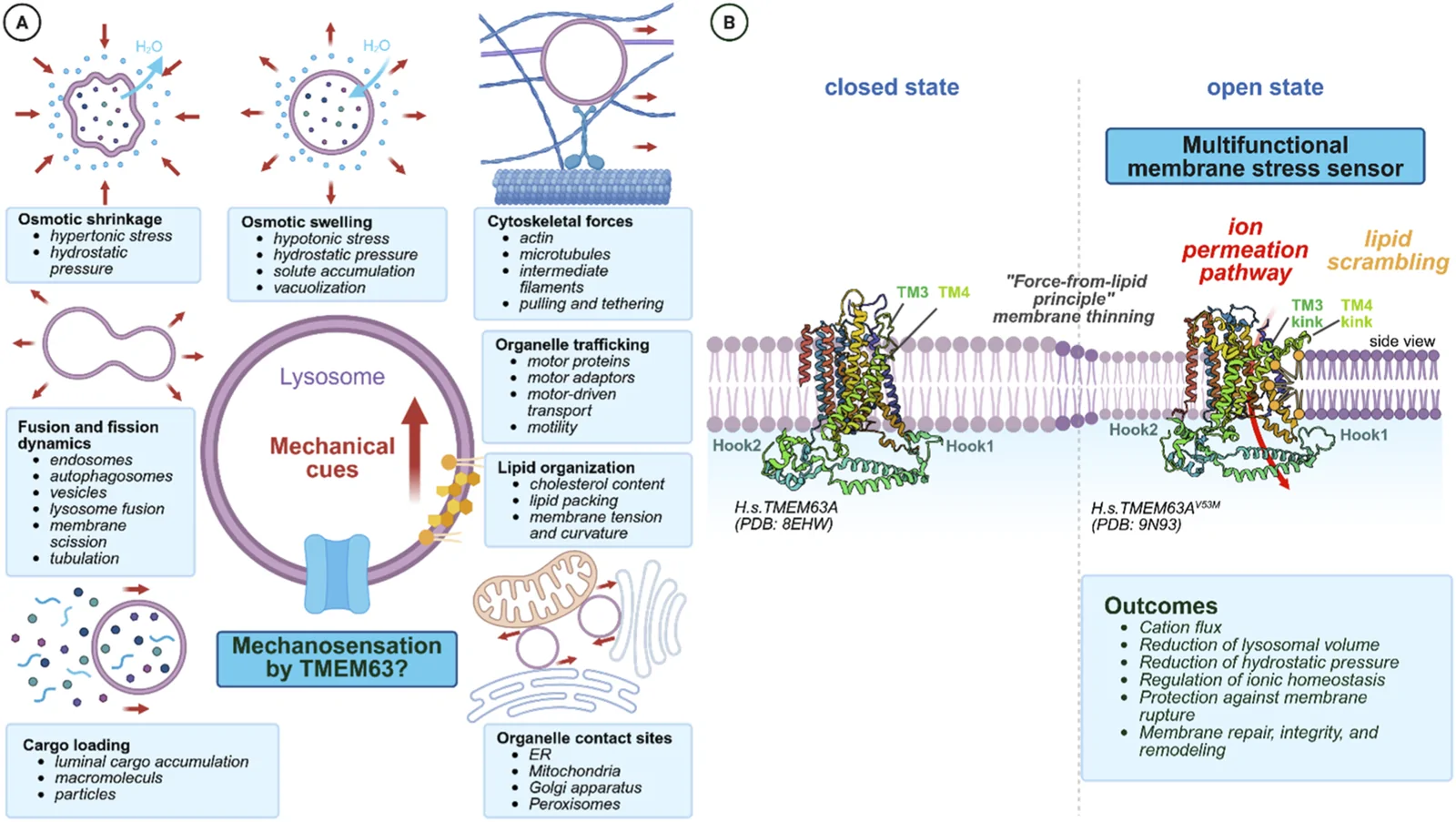
Proposed model of TMEM63-mediated mechanosensation. **(A)** Sources of mechanical stress on the lysosome-level. Lysosomes continuously experience diverse mechanical stimuli generated by changes in their physical and intracellular environment. These include osmotic shrinkage induced by hypertonic stress and osmotic swelling caused by hypotonic stress, hydrostatic pressure, solute accumulation, and vacuolization. Additional mechanical cues arise from membrane fusion and fission events associated with endosomes, autophagosomes and lysosomes, cargo loading, cytoskeletal pulling and tethering forces mediated by actin, microtubules and intermediate filaments, organelle trafficking driven by motor proteins, alterations in membrane lipid organization (including cholesterol content, lipid packing), and dynamic membrane contact sites with the endoplasmic reticulum (ER), mitochondria, Golgi apparatus and peroxisomes. Red arrows indicate putative mechanical cues (e.g., membrane tension, stretching, pulling, or hydrostatic pressure) that are proposed to contribute to TMEM63 activation. **(B)** Proposed structural model of TMEM63 activation. Under resting conditions, TMEM63 channels reside in a closed conformation. Mechanical stress and increased membrane tension are proposed to induce membrane thinning and bilayer deformation, resulting in conformational rearrangements involving the TM3 and TM4 helices. These structural transitions open the ion-conducting pore and permit cation permeation and recent structural studies additionally suggest activation of phospholipid scrambling activity. Whether ion conduction and lipid scrambling occur simultaneously within a common open state or represent distinct conformational states remains an important unresolved question. The structural models shown are based on the cryo-electron microscopy structures of the human TMEM63A in the closed state (PDB: 8EHW) and a constitutively active TMEM63A-V53M mutant representing an open-state conformation (PDB: 9N93). Activation of TMEM63 proteins is proposed to regulate lysosomal ion homeostasis, promote cation release, reduce hydrostatic pressure and lysosomal volume, facilitate membrane remodeling and repair, preserve membrane integrity, and coordinate adaptive responses to osmotic stress. Created with BioRender.com.

An additional conceptual advance has emerged from the discovery that TMEM63 proteins may possess functions extending beyond ion conduction (Figure 3B). Recent studies indicate that TMEM63 channels can act as mechanically regulated phospholipid scramblases (Figure 3B) linking membrane force sensing to lipid remodeling and membrane homeostasis (Zheng et al., 2025; Miyata et al., 2025; Lin et al., 2026).

Moreover, TMEM63A has recently been identified as a lysosomal mechanosensitive ion channel (Table 1), demonstrating that mechanosensation is not only restricted to the plasma membrane but can also occur within intracellular organelles (Chen et al., 2024; Tsvilovskyy et al., 2024; Li et al., 2024) (Tables 1 and 2). These findings have expanded the scope of TMEM63 biology and support an emerging view in which mechanosensitive signaling may potentially contribute to the regulation of intracellular membrane systems (Table 2).

**TABLE 1:** Current evidence for subcellular localization of TMEM63 proteins.

Subcellular compartment	TMEM63A	TMEM63B	TMEM63C	Representative cell types	Strength of evidence	References
Plasma membrane	+++	+++	++	Oligodendrocytes, alveolar type II (AT2) epithelial cells, dorsal root ganglion (DRG) neurons, subfornical organ (SFO) neurons, β-cells, podocytes	Strong	Murthy et al. (2018), Zhao et al. (2016), Zheng et al. (2023), Wang Y. Y. et al. (2025), Dereddi et al. (2026), Du et al. (2020), Zou et al. (2025), Chen et al. (2024)
Endosomes (Endolysosomal compartments)	+	?	?	Neurons, glia	Limited to moderate	Li et al. (2024)
Lysosomes	+++	?	?	Neurons, glia, lysosome associated compartments	Strong	Tsvilovskyy et al. (2024), Li et al. (2024)
Lysosome-related organelles (lamellar bodies)	+++	+++	—	Alveolar type II (AT2) epithelial cells	Strong	Chen et al. (2024)
Acidic secretory vesicles	+++	+	—	Pancreatic acinar cells	Strong for TMEM63A	Tsvilovskyy et al. (2024)
Endoplasmic reticulum (ER)	+	?	+++	Neurons	Strong for TMEM63C	Tábara et al. (2022), Zhang T. M. et al. (2023)
ER-mitochondria contact sites (MAMs)	—	—	+++	Neurons	Strong for TMEM63C	Tábara et al. (2022)
Golgi apparatus	—	—	—	No evidence currently available	—	—

Symbols indicate relative strength of localization evidence rather than protein abundance: +++, strong evidence; ++, moderate evidence; +, limited evidence; ?, unresolved; —, no evidence currently available.

**TABLE 2:** Cellular distribution, subcellular localization, physiological functions and disease associations of TMEM63 proteins.

Protein	Cell type	Tissue	Organ/system	Subcellular localization	Physiological function	Associated disease/pathology	References
TMEM63A	Oligodendrocytes	White matter	CNS (brain, spinal cord)	Plasma membrane; endosomal and lysosomal localization reported	Mechanosensitive Ca^2+^ signaling, oligodendrocyte differentiation, myelin sheath formation and maintenance	Hypomyelinating leukodystrophy-19 (HLD19)	Wang Y. Y. et al. (2025), Halford et al. (2025), Dereddi et al. (2026), Yan et al. (2019), Tonduti et al. (2021), Fukumura et al. (2022)
TMEM63A	Oligodendrocyte precursor cells (OPCs)	White matter	CNS	Plasma membrane	Mechanosensation during developmental myelination	Developmental hypomyelination	Wang Y. Y. et al. (2025), Dereddi et al. (2026)
TMEM63A	Non-peptidergic nociceptors	Dorsal root ganglion (DRG)	Peripheral nervous system	Plasma membrane	Mechanical pain sensation and inflammatory pain signaling	Chronic post-amputation pain	Pu et al. (2023)
TMEM63A	Alveolar type II (AT2) epithelial cells	Alveolar epithelium	Lung	Lamellar bodies (lysosome-related organelles); plasma membrane	Stretch-induced surfactant secretion during lung inflation	Respiratory failure and atelectasis in knockout mice	Chen et al., 2024; Hook (2024)
TMEM63A	Pancreatic acinar cells	Exocrine pancreas	Pancreas	Acidic secretory vesicles (lysosome-related organelles)	Regulation of Ca^2+^ release from acidic secretory vesicles and exocytosis	Increased susceptibility to acute and chronic pancreatitis	Tsvilovskyy et al. (2024), Patel and Yule (2024)
TMEM63A	Breast cancer cells	Mammary epithelium	Breast	Not fully resolved	Cell proliferation, migration, invasion, and stress adaptation	Triple-negative breast cancer (TNBC) progression	Zhang T. M. et al. (2023)
TMEM63B	Alveolar type II (AT2) epithelial cells	Alveolar epithelium	Lung	Lamellar bodies	Surfactant secretion during lung inflation	Respiratory insufficiency in knockout mice	Chen et al., 2024; Hook (2024)
TMEM63B	Inner hair cells	Organ of Corti	Cochlea	Plasma membrane	Hypoosmotic Ca^2+^ influx and hearing function	Progressive hearing loss and deafness	Du et al. (2020)
TMEM63B	Cochlear sensory epithelial cells	Cochlear sensory epithelium	Inner ear	Plasma membrane	Cochlear maturation and auditory development	Auditory dysfunction	Ye et al. (2024)
TMEM63B	Excitatory neurons	Subfornical organ (SFO)	Brain	Plasma membrane	Hyperosmotic sensing and thirst regulation	Impaired thirst responses	Yang et al. (2024), Zou et al. (2025)
TMEM63B	Migrating olfactory interneurons	Olfactory bulb	Brain	Plasma membrane	Membrane tension sensing, Ca^2+^ signaling, and somal translocation during neuronal migration	Defective neuronal migration	Minegishi et al. (2025)
TMEM63B	Pancreatic β-cells	Islets of Langerhans	Pancreas	Plasma membrane	Regulation of glucose-stimulated insulin secretion	Impaired insulin secretion	Tu et al. (2025)
TMEM63B	Intestinal stem cells (ISCs)	Intestinal crypts	Intestine	Plasma membrane	Regulation of stem-cell proliferation, epithelial renewal and intestinal motility	Increased dextran sulfate sodium (DSS)-induced colitis severity in mice	Tu et al. (2024)
TMEM63B	Cortical and subcortical neurons	CNS	Brain	Plasma membrane	Neuronal osmosensation, membrane excitability, neurodevelopment and osmotic homeostasis	Developmental and epileptic encephalopathy (DEE)	Yang et al. (2024), Zou et al. (2025), Sasaki et al. (2024), Vetro et al. (2023)
TMEM63C	Podocytes	Glomerular filtration barrier	Kidney	Plasma membrane	Maintenance of podocyte integrity and filtration barrier function	Albuminuria and focal segmental glomerulosclerosis (FSGS)	Orphal et al. (2020), Schulz et al. (2019)
TMEM63C	Podocytes	Glomerulus	Kidney	Plasma membrane	Protection against Angiotensin II induced injury and apoptosis	Chronic kidney disease progression	Eisenreich et al. (2020)
TMEM63C	Neurons	CNS	Brain and spinal cord	Endoplasmic reticulum (ER); ER-mitochondria contact sites (MAMs)	Regulation of organelle morphology, phospholipid homeostasis and ER- mitochondria communication	Hereditary spastic paraplegia (HSP)	Tábara et al. (2022)

Lysosomes are increasingly recognized as signaling organelles that regulate calcium homeostasis, membrane trafficking, autophagy, nutrient sensing and cellular adaptation to stress response through a diverse repertoire of resident ion channels and transporters (Kondratskyi et al., 2025; Kondratskyi et al., 2018; Xu and Ren, 2015; Li et al., 2019; Lloyd-Evans and Waller-Evans, 2020). The discovery that TMEM63 channels can directly sense membrane tension has introduced a new dimension to lysosomal ion channel biology and suggests that intracellular organelles actively participate in mechanosignaling pathways. These findings place TMEM63 proteins within the broader framework of lysosomal signaling networks and organellar mechanobiology (Figure 3A).

In this review, we summarize current knowledge of the TMEM63/OSCA family with particular emphasis on their structural organization, gating mechanisms, and emerging roles as mechanosensitive ion channels in both plasma membrane and intracellular compartments (Tables 1 and 2). Special attention is given to recent discoveries identifying TMEM63 channels as lysosomal mechanosensitive ion channels and to the broader concept of lysosomal mechanotransduction (Figure 3A). We discuss how TMEM63-mediated signaling integrates with established lysosomal ion channel networks, including TRPML, TPC and TMEM175 channels, to regulate organellar homeostasis, stress adaptation, and intracellular signaling. Furthermore, we examine the physiological functions and disease associations of TMEM63 family members and discuss emerging evidence linking mechanosensation, lipid remodeling, and lysosomal signaling. Finally, we highlight outstanding questions and future directions that may establish TMEM63 proteins as regulators of lysosomal mechanobiology and promising therapeutic targets in neurological, renal, pulmonary, metabolic and lysosome-associated disorders.

## 2 Mechanosensitive ion channels: general principles

Mechanosensitive ion channels are specialized membrane proteins that convert mechanical stimuli into electrochemical signals. By coupling membrane deformation to ion flux, these channels enable cells to rapidly sense and adapt to physical forces. Mechanically activated currents have been identified in organisms ranging from bacteria to mammals, highlighting the evolutionary importance of mechanosensation and mechanotransduction as fundamental biological processes (Kefauver et al., 2020; Poole, 2022; Martinac et al., 1990; Cox et al., 2019; Martinac and Kloda, 2003).

Cells experience a variety of mechanical stimuli, including membrane stretch, hydrostatic pressure, shear stress, osmotic swelling, extracellular matrix stiffness, and tissue deformation (Kefauver et al., 2020; Poole, 2022; Douguet and Honore, 2019; Di et al., 2023; Jin et al., 2020; Gu and Gu, 2014). These forces alter membrane tension, curvature, lipid organization, and cytoskeletal architecture, thereby influencing membrane protein conformation. Opening of mechanosensitive ion channels permits fluxes of Ca^2+^, Na^+^, K^+^ and Cl^−^, which regulate membrane potential, calcium signaling, excitability, secretion, proliferation, differentiation, migration, and survival (Kefauver et al., 2020; Poole, 2022; Douguet and Honore, 2019; Di et al., 2023; Murthy et al., 2017; Jin et al., 2020; Gu and Gu, 2014).

Two major concepts explain how mechanical forces activate ion channels: In the force-from-lipids model (Pliotas et al., 2015; Bavi et al., 2023; Martinac et al., 1990; Perozo et al., 2002; Jin et al., 2020; Kocer, 2015; Teng et al., 2015; Anishkin et al., 2014), mechanical stress is transmitted directly through the lipid bilayer, where changes in membrane tension, thickness, curvature or lateral pressure profiles alter ion channel conformation and channel gating without requiring additional accessory proteins (Teng et al., 2015; Anishkin et al., 2014; Pliotas et al., 2015; Bavi et al., 2023; Martinac et al., 1990; Perozo et al., 2002; Brohawn, 2015). This mechanism was first established through studies of the bacterial mechanosensitive channels MscL and MscS, which remain mechanosensitive when reconstituted into artificial lipid membranes (Martinac et al., 1990; Perozo et al., 2002; Cox et al., 2018; Nomura et al., 2012), and later, this concept was extended to eukaryotic channels, such as PIEZO (Murthy et al., 2017; Coste et al., 2010; Syeda, 2021; Wu et al., 2017; Xiao, 2024) and two-pore domain potassium channels (K2P) (Brohawn, 2015; Brohawn et al., 2014; Honore, 2007; Patel and Honore, 2001). In the complementary force-from-filament or force-from-tether model, mechanical forces are transmitted through the cytoskeleton, extracellular matrix, or by accessory proteins that can even assemble into large ion channel complexes (Arnadottir and Chalfie, 2010; Anishkin et al., 2014; Jin et al., 2020; Brohawn, 2015; Goldmann, 2012; Kuo, 2013; Geiger et al., 2009; Jia et al., 2020). These mechanisms are not mutually exclusive, and individual channels may integrate lipid-mediated and protein-mediated forces depending on their cellular context.

It is also important to distinguish directly mechanosensitive ion channels from ion channels that act downstream of other mechanosensors. Mechanical stimuli can activate G-protein coupled receptors (GPCRs), integrins, receptor tyrosine kinases, or cytoskeletal signaling complexes, which then secondarily activate ion channels through intracellular signaling cascades (Di et al., 2023; Mederos y Schnitzler et al., 2011; Storch et al., 2012; Martino et al., 2018; Erdogmus et al., 2019). Thus, the term “mechanosensitive” may refer either to direct force sensing by the channel itself or to the participation of a channel in a broader mechanotransduction pathway.

Mechanosensitivity has evolved independently in multiple ion channel families, resulting in remarkable structural and functional diversity (Kefauver et al., 2020; Poole, 2022; Murthy et al., 2017; Jin et al., 2020). The best-characterized mammalian mechanosensitive channels are the PIEZO channel family with PIEZO1 and PIEZO2, that mediate touch sensation, proprioception, vascular development, erythrocyte volume regulation, and numerous other physiological functions (Murthy et al., 2017; Coste et al., 2010; Syeda, 2021; Wu et al., 2017).

Although mechanosensation and mechanotransduction have traditionally been investigated at the plasma membrane, growing evidence suggests that intracellular organelles are also susceptive to mechanical cues (Figure 3A). Lysosomes, endosomes, secretory granules, and related organelles experience continuous changes in membrane tension, curvature, luminal volume, osmotic pressure, and lipid composition during trafficking, degradation, secretion, and stress adaptation (Kondratskyi et al., 2025; Kondratskyi et al., 2018; Xu and Ren, 2015; Li et al., 2019; Lloyd-Evans and Waller-Evans, 2020; Hu et al., 2022a; Yu and Yang, 2019; Radulovic et al., 2026; Jain and Zoncu, 2026; Ballabio and Bonifacino, 2020; Dong et al., 2010; Venkatachalam et al., 2015; Grimm et al., 2017). Lysosomes in particular contain a complex repertoire of ion channels and transporters that regulate calcium signaling, membrane potential, pH, volume, trafficking, autophagy, and stress responses. The identification of TMEM63A as a lysosomal mechanosensitive ion channel has therefore expanded the concept of mechanotransduction beyond the plasma membrane and suggests that force sensing may be a general property of biological membranes (Li et al., 2024).

Within this broader mechanobiological landscape, the OSCA/TMEM63 family occupies a unique position at the interface between mechanosensitive ion conduction, membrane lipid organization, and phospholipid scrambling (Figure 3B).

## 3 Discovery and classification of the TMEM63/OSCA family

The TMEM63/OSCA family represents one of the largest and most evolutionarily conserved groups of mechanosensitive membrane proteins in eukaryotes (Figure 1) (Murthy et al., 2018). Members of this family were initially identified in plants as hyperosmolarity-activated calcium-permeable channels involved in osmotic stress responses and were subsequently recognized as the mammalian homologs specifically of the Arabidopsis Reduced Hyperosmolality-Induced Calcium Increase (OSCA) proteins (Murthy et al., 2018; Hou et al., 2014; Yuan et al., 2014). The broad phylogenetic distribution of OSCA/TMEM63 proteins across plants, invertebrates, and vertebrates (Figure 1) suggests that the ability to sense membrane deformation and osmotic stress is an ancient cellular function that emerged early during eukaryotic evolution (Murthy et al., 2018; Hou et al., 2014).

Phylogenetic analyses indicate substantial diversification of the family during evolution (Figure 1). Plant genomes often encode expanded OSCA repertoires. For example, *Arabidopsis thaliana* encodes 15 OSCA proteins, whereas Oryza sativa and Zea mays possess 11 and 12 homologs, respectively. These proteins can be grouped into several subfamilies with distinct expression patterns and physiological functions (Murthy et al., 2018; Hou et al., 2014; Yuan et al., 2014; Cao et al., 2020). In contrast, vertebrates typically express three paralogous genes (TMEM63A, TMEM63B and TMEM63C), whereas many invertebrates contain only a single ortholog, exemplified by *Drosophila melanogaster* with DmTMEM63 (Murthy et al., 2018; Zheng et al., 2023; Li et al., 2024) (Figure 1). Despite this diversification, the overall domain architecture and mechanosensitive properties remain remarkably conserved (Figure 1), highlighting the evolutionary importance of membrane-based force sensing.

Major advances in cryo-EM have transformed our understanding of TMEM63/OSCA channel structure (Zheng et al., 2023; Jojoa-Cruz et al., 2018; Liu et al., 2018; Zhang M. et al., 2018; Maity et al., 2019; Jojoa-Cruz et al., 2024; Zhang M. et al., 2023; Qin et al., 2023; Han et al., 2024; Wu et al., 2024). Initial structures of plant OSCA channels revealed homodimeric assemblies in which each subunit contains eleven transmembrane helices and forms an independent ion-conducting pore (Jojoa-Cruz et al., 2018; Liu et al., 2018; Zhang M. et al., 2018; Maity et al., 2019; Jojoa-Cruz et al., 2024). The permeation pathway is primarily formed by transmembrane helices TM3 to TM7, generating a dual-pore architecture with structural similarities to members of the TMEM16 (Brunner et al., 2014; Pedemonte and Galietta, 2014) and TMC protein families (Pan et al., 2018). Subsequent structural studies demonstrated that the dimeric organization is stabilized by extensive interactions within intracellular linker regions and transmembrane interfaces (Zhang M. et al., 2018; Maity et al., 2019). However, mammalian TMEM63 channels are predicted to function as monomeric, high-threshold, single-pore conduits according to cryo-EM analysis (Zhao et al., 2016; Zheng et al., 2023; Zhang M. et al., 2023; Qin et al., 2023; Schulz et al., 2019; Wu et al., 2024).

More recent studies have shown that mammalian TMEM63 proteins exhibit notable structural divergence from their plant counterparts. Cryo-EM structures of TMEM63 proteins indicate that mammalian channels predominantly adopt monomeric conformations while preserving the overall OSCA/TMEM63 fold (Zheng et al., 2023; Qin et al., 2023; Wu et al., 2024). Structural and functional analysis suggest that alterations within the intracellular linker 2 (IL2) region reduce the tendency for stable dimerization and promote monomeric channel organization (Zheng et al., 2023; Qin et al., 2023; Wu et al., 2024). Electrophysiological studies further demonstrated that monomeric TMEM63 proteins retain full mechanosensitive channel activity, establishing that a single subunit is sufficient to form a functional mechanosensitive pore (Zheng et al., 2023; Qin et al., 2023; Wu et al., 2024).

A defining structural feature of both plant and mammalian family members is the presence of a large membrane-facing cavity and lipid-accessible cleft closely associated with pore-forming helices (Zheng et al., 2023; Zhang M. et al., 2018; Maity et al., 2019; Zhang M. et al., 2023). This architecture places membrane lipids in direct contact with gating elements and provides a structural basis for force-from-lipid activation mechanism. Consistent with this concept, multiple cryo-EM structures revealed lipid-like densities occupying positions within the pore region and along transmembrane interfaces, indicating that lipid-protein interactions are integral to channel architecture and function rather than passive features of the membrane environment (Zheng et al., 2023; Zhang M. et al., 2018; Maity et al., 2019; Zhang M. et al., 2023; Han et al., 2024).

Recent studies have provided unprecedented insights into the conformational changes underlying channel activation. Open-state structures of OSCA channels demonstrated that membrane tension induces substantial rearrangements of pore-lining helices and associated lipid-interacting domains, resulting in expansion of the ion-conducting pathway (Zhang M. et al., 2023; Han et al., 2024; Shan et al., 2024). In particular, mechanical force appears to be transmitted through membrane-embedded helices that function as structural sensors of bilayer tension, thereby coupling membrane deformation directly to channel gating (Zhang M. et al., 2023; Han et al., 2024; Shan et al., 2024), which supports a *force-from-lipid gating mechanism* and establishes membrane mechanics as a central determinant of TMEM63/OSCA channel activation.

Collectively, current structural evidence positions TMEM63/OSCA proteins as a distinct class of mechanosensitive membrane proteins whose activity is linked to membrane organization, lipid composition, and bilayer mechanics.

## 4 Structural organization and activation of TMEM63/OSCA channels

### 4.1 Mechanisms of mechanical gating of TMEM63/OSCA channels

As mentioned, current evidence indicates that members of the TMEM63/OSCA family are primarily activated through a *force-from-lipid mechanism*, in which mechanical forces are transmitted directly through the lipid bilayer rather than through accessory tethering proteins (Teng et al., 2015; Anishkin et al., 2014; Pliotas et al., 2015; Bavi et al., 2023; Martinac et al., 1990; Perozo et al., 2002; Brohawn, 2015). In this model, changes in membrane tension, curvature, lateral pressure or lipid packing alter protein-lipid interactions, thereby inducing structural rearrangements that promote channel mechanogating. The structural basis for this mechanism has been elucidated through a series of cryo-EM studies of plant OSCA channels and mammalian TMEM63 proteins (Zheng et al., 2023; Jojoa-Cruz et al., 2018; Liu et al., 2018; Zhang M. et al., 2018; Maity et al., 2019; Jojoa-Cruz et al., 2024; Zhang M. et al., 2023; Qin et al., 2023; Han et al., 2024; Shan et al., 2024; Wu et al., 2024). Early structural analyses revealed that OSCA channels contain extensive membrane-facing surfaces and lipid accessible fenestrations that position the channel in close contact with the surrounding bilayer (Jojoa-Cruz et al., 2018; Liu et al., 2018; Zhang M. et al., 2018; Jojoa-Cruz et al., 2024). These observations suggest that membrane lipids are integral components of the gating apparatus that supports direct mechanogating.

A major advance in understanding channel mechanogating came from the identification of a mechanical-coupling mechanism in which membrane tension is translated to conformational changes within the transmembrane domain, which are then conveyed to the pore-forming region (Zhang M. et al., 2023). Structural comparisons between closed and open states demonstrated that mechanical stimulation is associated with large-scale rearrangements of transmembrane helices and displacement of lipid molecules occupying the dimer interface and pore neighboring cavities. In this model, lipid molecules act as stabilizing elements of the closed state, and force-induced lipid displacement facilitates pore opening and ion permeation (Zhang M. et al., 2023). Similar principles have been proposed for bacterial mechanosensitive channels such as MscL and MscS, supporting the notion that *force-from-lipid activation* represents an evolutionarily conserved mechanism of intrinsic mechanosensitivity (Bavi et al., 2023; Perozo et al., 2002; Cox et al., 2019; Martinac and Kloda, 2003; Cox et al., 2018; Nomura et al., 2012). Subsequent cryo-EM studies captured multiple intermediate and conducting conformations of OSCA channels and refined our understanding of the gating process (Han et al., 2024; Shan et al., 2024).

In detail, channel activation involves coordinated movements of several transmembrane helices, particularly of the transmembrane domain 6 (TM6) and neighboring pore lining regions, together with extensive remodeling of membrane-associated lipids. Channel opening is accompanied by progressive extrusion of lipids from the permeation pathway, dilation of extracellular pore constrictions, monomer contraction, and membrane thinning with reorganization of the dimer interface. Full activation requires not only extracellular dilation, which is referred to as a blooming‐like channel opening, but also intracellular pore opening, driven by an upward movement of the TM6 (Shan et al., 2024). Importantly, these findings demonstrate that lipids actively participate in the gating process rather than serving merely as a structural environment. Recent structural work has further led to the proposal of a contract-to-open model for OSCA channel activation (Han et al., 2024). In this framework, membrane tension induces contraction of the dimer interface with extrusion of intracellular lipids, which in turn permits outward expansion of individual pore-forming domains. This conformational transition creates a lateral opening toward the surrounding membrane and generates a proteolipidic permeation pathway in which both protein residues and membrane lipids contribute to ion conduction (Han et al., 2024). This mechanism is essential for channel opening, and in the process, each subunit can open independently, as evidenced by asymmetric open-state conformations. Notably, individual subunits can adopt distinct conformational states, suggesting that the two pores within dimeric plant OSCA channels may operate semi-independently and potentially exhibit asynchronous gating behavior (Han et al., 2024; Shan et al., 2024).

Despite differences in structural interpretation, current models converge on several key principles. First, membrane lipids are active determinants of channel gating rather than passive structural components. Second, mechanical activation requires force-induced remodeling of lipid-protein interactions within membrane-embedded regions of the channel. Third, pore opening involves coordinated conformational rearrangements that directly couple membrane deformation to ion conduction. Collectively, these findings establish TMEM63/OSCA proteins as prototypical force-from-lipid mechanosensors that directly monitor the physical state of the surrounding membrane (Zhang M. et al., 2023; Han et al., 2024; Shan et al., 2024; Teng et al., 2015; Anishkin et al., 2014; Pliotas et al., 2015; Bavi et al., 2023; Martinac et al., 1990; Perozo et al., 2002).

However, the mechanogating mechanisms of mammalian TMEM63 proteins are not fully understood because high-resolution structures of the open state induced by mechanical forces have not yet been captured. Nevertheless, electrophysiological and mutagenesis studies have identified critical residues within TM6 and the IL2 region that are required for mechanically evoked currents (Zheng et al., 2023; Zheng et al., 2025). Recent structures of TMEM63A and TMEM63C indicate that mammalian channels predominantly function as monomers while preserving the core mechanosensitive architecture observed in plant OSCA channels (Zheng et al., 2023; Qin et al., 2023; Wu et al., 2024). These observations suggest that fundamental force-from-lipid principles are conserved throughout the TMEM63/OSCA family, although species-specific adaptations may modulate activation thresholds, ion selectivity and gating kinetics (Zheng et al., 2023; Zhang M. et al., 2023; Zheng et al., 2025).

Emerging evidence further indicates that membrane composition represents an important determinant of TMEM63 channel activity. Cholesterol content, lipid packing and membrane mechanical properties influence mechanosensitive activation, suggesting that TMEM63 proteins function not only as force sensors but also as detectors of membrane organization (Miyata et al., 2025; Lin et al., 2026). Such properties may be particularly relevant in intracellular organelles, where lipid composition and membrane curvature differ substantially from those of the plasma membrane. Future structural studies capturing mechanically activated mammalian TMEM63 channels in defined lipid environments will therefore be essential to fully understand how membrane mechanics regulate TMEM63 channel gating in physiological and pathological settings.

### 4.2 TMEM63 proteins as mechanosensitive lipid scramblases

Recent discoveries have substantially expanded the functional repertoire of TMEM63 proteins beyond their established role as mechanosensitive ion channels. Structural, biochemical, and cellular studies now indicate that selected members of the TMEM63 family can also function as mechanically regulated phospholipid scramblases (Figure 3B), revealing an unexpected link between mechanosensation, membrane remodeling, mechanotransduction and lipid homeostasis (Vetro et al., 2023; Miyata et al., 2025; Lin et al., 2026). These findings challenge the traditional view of TMEM63 proteins as exclusively ion-conducting channels and suggest that they may represent a unique class of multifunctional membrane stress sensors (Figure 3B).

Phospholipid scrambling refers to the bidirectional translocation of phospholipids between the inner and outer leaflets of biological membranes. Under physiological conditions, cellular membranes maintain a highly asymmetric lipid distribution through the coordinated activity of flippases, floppases, and scramblases. This asymmetry is essential for membrane identity, intracellular trafficking, signal transduction, membrane curvature generation, and cell survival. Disruption of lipid asymmetry can profoundly alter membrane biophysical properties and cellular signaling pathways (Brunner et al., 2014; Pedemonte and Galietta, 2014; Wang H. Y. et al., 2025). While phospholipid scrambling is classically associated with calcium-activated scramblases such as proteins of the TMEM16 family, recent studies indicate that membrane mechanics can also regulate lipid translocation pathways (Brunner et al., 2014; Pedemonte and Galietta, 2014; Wang H. Y. et al., 2025).

The first evidence linking TMEM63 proteins to phospholipid scrambling was provided by structural and functional studies of TMEM63B (Miyata et al., 2025). Using cryo-EM, lipid-translocation assays, and membrane perturbation experiments, Miyata and colleagues demonstrated that TMEM63B possesses membrane structure-responsive scramblase activity that is activated by alterations in membrane organization (Miyata et al., 2025). Changes in membrane curvature, bilayer thickness or cholesterol content induced conformational rearrangements that exposed a lateral hydrophilic groove connecting the two membrane leaflets. This pathway provides a favorable environment for phospholipid headgroup translocation across the hydrophobic core of the bilayer and resembles the lipid-conduction pathways previously described for TMEM16 scramblases (Miyata et al., 2025; Brunner et al., 2014). Importantly, TMEM63B-mediated scrambling occurred independently of classical calcium-dependent activation mechanisms, suggesting that the physical properties of the membrane can themselves serve as regulatory stimuli (Miyata et al., 2025).

Subsequent studies further established a direct relationship between mechanical cues and TMEM63-dependent lipid remodeling (Lin et al., 2026). Lin and colleagues demonstrated that membrane stretch and mechanical deformation promote phospholipid scrambling by TMEM63 proteins and identified cholesterol as a critical negative regulator of this process that stabilizes the closed conformation and inhibits lipid scrambling through the open pore (Lin et al., 2026).

Structural analyses revealed that cholesterol molecules occupy membrane accessible regions associated with the scrambling pathway, thereby stabilizing conformations with reduced lipid translocation activity. Conversely, cholesterol depletion enhanced scrambling efficiency and increased membrane plasticity (Lin et al., 2026). These observations indicate that TMEM63 proteins are sensitive not only to mechanical force but also to membrane organization, suggesting that they function as integrated sensors of membrane mechanical state and lipid organization.

Functional studies further demonstrated that TMEM63-dependent lipid scrambling contributes to cellular adaptation during mechanical stress (Lin et al., 2026). Cells lacking TMEM63 proteins exhibited increased susceptibility to membrane rupture and reduced survival under conditions of mechanical challenge, whereas TMEM63-mediated scrambling promoted membrane resilience and facilitated restoration of membrane integrity (Lin et al., 2026). These findings suggest that lipid translocation may represent an adaptive response that enables membranes to accommodate force-induced changes in curvature, tension and surface area.

Additional support for a physiologically relevant scramblase function has emerged from the characterization of disease-associated TMEM63 variants (Zheng et al., 2025; Vetro et al., 2023; Miyata et al., 2025; Lin et al., 2026). A recurrent TMEM63B-V44M mutation identified in patients with developmental and epileptic encephalopathy produces constitutive scramblase activity, disrupting membrane lipid asymmetry and linking TMEM63B dysfunction to neurodevelopmental disorders (Vetro et al., 2023; Miyata et al., 2025). Similarly, the homologous TMEM63A-V53M variant promotes lipid translocation through the formation of a membrane accessible lateral cleft while exerting comparatively modest effects on mechanically activated ion currents (Zheng et al., 2025). These observations suggest that disease pathogenesis may arise not only from altered ion channel activity but also from dysregulated membrane remodeling and disturbed phospholipid homeostasis.

Despite these advances, the mechanistic relationship between ion conduction and lipid scrambling remains incompletely understood. One unresolved question is whether these activities occur concurrently within the same conformational state or instead correspond to distinct functional states of the protein. Structural studies suggest that conformational rearrangements associated with mechanical activation expose membrane-facing fenestrations that could potentially support both ion permeation and lipid translocation (Han et al., 2024; Miyata et al., 2025; Lin et al., 2026). However, whether ion conduction and scrambling are mechanistically coupled or independently regulated remains unknown. It is also possible that different TMEM63 family members exhibit varying degrees of specialization, with some isoforms functioning predominantly as mechanosensitive ion channels and others displaying more pronounced lipid scrambling activity.

Another important unresolved issue concerns the physiological relevance of TMEM63-dependent scrambling within intracellular organelles. Given the recent identification of TMEM63A in lysosomal membranes (Li et al., 2024), it is conceivable that mechanically regulated phospholipid translocation contributes to organellar membrane remodeling during lysosome swelling under osmotic pressure, vesicle fusion and fission dynamics, cargo loading, membrane repair, and osmotic adaptation (Figure 3A). Such a mechanism would provide a new conceptual framework linking intracellular mechanosensation to membrane homeostasis, although direct experimental evidence remains limited.

Collectively, current evidence supports a possible model in which TMEM63 proteins function at the interface of mechanosensation and lipid scrambling. Beyond acting as mechanosensitive ion channels, TMEM63 proteins appear capable of translating changes in membrane tension, curvature, and lipid composition into coordinated regulation of both ion flux and phospholipid distribution (Vetro et al., 2023; Miyata et al., 2025; Lin et al., 2026). This emerging dual-function paradigm substantially broadens the biological significance of the TMEM63 family and establishes these proteins as central regulators of membrane adaptation to mechanical stress. Future studies capturing mechanically activated mammalian TMEM63 structures and elucidating the molecular relationship between ion conduction and lipid scrambling will be essential for understanding how these processes contribute to physiology and disease.

### 4.3 Intracellular mechanosensation and lysosomal ion channel signaling

Although mechanosensation has traditionally been studied at the plasma membrane, accumulating evidence suggests that intracellular organelles also possess mechanosensitive capabilities. Among these organelles, lysosomes have emerged as dynamic signaling hubs that integrate mechanical, osmotic, metabolic, and biochemical cues to maintain cellular homeostasis. Beyond their classical role as degradative compartments, lysosomes regulate calcium signaling, membrane trafficking, autophagy, nutrient sensing, and cellular stress responses through a diverse repertoire of ion channels and transporters embedded within the lysosomal membrane (Kondratskyi et al., 2025; Xu and Ren, 2015; Li et al., 2019; Lloyd-Evans and Waller-Evans, 2020; Radulovic et al., 2026). Consequently, lysosomes are increasingly viewed not merely as recycling compartments of the cell but as active signaling organelles capable of coordinating adaptive cellular responses to changing environmental conditions (Xu and Ren, 2015; Hu et al., 2022a; Jain and Zoncu, 2026; Ballabio and Bonifacino, 2020).

The lysosomal membrane is continuously exposed to mechanical forces (Figure 3A) generated by osmotic fluctuations, solute and cargo accumulation, membrane fusion and fission events, intracellular transport, lipid organization and interactions with the cytoskeleton (Kondratskyi et al., 2025; Xu and Ren, 2015; Li et al., 2019; Lloyd-Evans and Waller-Evans, 2020; Hu et al., 2022a; Radulovic et al., 2026). These processes dynamically alter membrane tension, curvature, and lipid organization, creating a mechanical environment capable of influencing membrane proteins and ion channel activity. Such observations have led to the concept that lysosomes function as intracellular mechanically dynamic hubs that convert physical perturbations of organellar membranes into biochemical and ionic signals. This concept substantially expands the traditional view of mechanosensation by suggesting that force sensing is not restricted to the cell surface but may represent a general property of all biological membranes.

A major advance in this field was the identification of TMEM63A as a lysosomal mechanosensitive ion channel (Li et al., 2024). Using electrophysiological recordings from enlarged lysosomes, Li and colleagues demonstrated that both *Drosophila* TMEM63 and mammalian TMEM63A are directly activated by membrane stretch within lysosomal membranes (Li et al., 2024). Mechanical stimulation generated non-selective cation currents and promoted lysosomal calcium release, providing direct evidence that lysosomes can detect membrane tension through force-gated ion channels (Li et al., 2024). Furthermore, genetic deletion of TMEM63A markedly reduced lysosomal mechanosensitivity and impaired cellular adaptation to osmotic stress, indicating that intracellular force sensing by TMEM63 proteins is evolutionarily conserved (Li et al., 2024). These findings significantly broaden the functional repertoire of the TMEM63 family beyond plasma membrane mechanosensation and establish TMEM63 proteins as mechanosensitive ion channels that operate within an intracellular organelle.

Recent work by Kim et al. (2026) further refined this concept by identifying TMEM63A as a rapid lysosomal mechano-protective mechanism that relieves acute increases in lysosomal membrane tension. In this model, elevated hydrostatic pressure, osmotic swelling, lysosomotropic agents, or pathogen-induced luminal deformation increase tension within the lysosomal limiting membrane. Acute TMEM63A activation promotes efflux of monovalent cations from the lysosomal lumen, thereby reducing the luminal osmotic load. This cation efflux is proposed to cooperate with anion fluxes, including LRRC8/VRAC-dependent chloride movement, and is followed by water efflux, leading to reduced lysosomal volume, hydrostatic pressure, and membrane tension (Hu et al., 2022a; Kim et al., 2026; Li et al., 2020). Thus, TMEM63A may function as a lysosomal pressure-relief valve that protects the organelle from rupture and provides time for slower adaptive mechanisms, including homotypic fusion between lysosomes and lipid transfer from the endoplasmic reticulum, to increase membrane availability (Kim et al., 2026).

This mechanism is pathophysiologically relevant because loss of TMEM63A sensitizes lysosomes to acute mechanical and osmotic challenges, including hypotonic shock, lysosomotropic agents, and pathogen-induced phagolysosomal deformation during *Candida albicans* hyphal growth (Kim et al., 2026). Importantly, re-expression of wild-type TMEM63A, but not disease-associated loss-of-function variants, rescued lysosomal damage in TMEM63A-deficient cells, linking TMEM63A-dependent lysosomal mechanoresilience to human TMEM63A channelopathies (Yoneno et al., 2024; Yan et al., 2019; Tonduti et al., 2021; Fukumura et al., 2022; Kim et al., 2026). Together, these findings establish lysosomes as organelles with intrinsic mechano-regulatory capacity and position TMEM63A as a direct mediator of lysosomal membrane tension homeostasis (Figure 3).

The discovery of TMEM63A-mediated lysosomal mechanosensation challenges the long-standing assumption that mechanosensation and mechanotransduction occur primarily at the plasma membrane. However, intracellular membranes appear capable of continuously monitoring their own physical state and of translating alterations in membrane tension into signaling responses that preserve organellar and cellular homeostasis (Lin et al., 2026). Such a mechanism is particularly relevant for lysosomes, whose morphology and membrane properties are constantly modified by fluctuations in luminal content, membrane trafficking and cellular stress (Figure 3A).

Mechanically evoked lysosomal signaling is likely integrated within a broader network of lysosomal ion channels and transporters that collectively regulate organellar excitability and calcium homeostasis (Kondratskyi et al., 2025; Xu and Ren, 2015; Li et al., 2019; Lloyd-Evans and Waller-Evans, 2020; Radulovic et al., 2026). Among these, TRPML1 serves as the principal lysosomal calcium release channel that is activated by phosphatidylinositol-3,5-bisphosphate [PI(3,5)P_2_] and modulated by luminal pH and redox state (Dong et al., 2010; Venkatachalam et al., 2015; Wang et al., 2014). TRPML1-mediated calcium release controls membrane trafficking, lysosome autophagosome fusion, lysosomal exocytosis, organelle positioning, and autophagic flux (Li et al., 2019; Venkatachalam et al., 2015; Wang et al., 2014; Cao et al., 2017). Loss-of-function mutations in TRPML1 cause mucolipidosis type IV (MLIV), a lysosomal storage disorder characterized by impaired trafficking, defective autophagy, and neurodegeneration, highlighting the physiological importance of properly tuned lysosomal calcium signaling (Xu and Ren, 2015; Li et al., 2019). Although TRPML1 is not generally classified as an inherently mechanosensitive ion channel, its activity is strongly influenced by the composition of the membrane lipids and by phosphoinositide signaling, and the physical properties of the bilayer influence TRPML channel gating (Li et al., 2019; Venkatachalam et al., 2015; Wang et al., 2014; Cao et al., 2017; Liu and Montell, 2015). Mechanical changes in membrane tension, curvature or lipid organization may therefore indirectly modulate TRPML1-dependent signaling pathways, raising the possibility that TMEM63A and TRPML1 may operate within interconnected mechanochemical signaling networks (Gu and Xu, 2016; Zhang X. et al., 2018).

Lysosomal membrane excitability is further shaped by additional ion channels that regulate membrane potential and ionic homeostasis. Two-pore channels (TPC1 and TPC2) function as sodium-permeable channels that contribute to endolysosomal excitability and vesicular trafficking (Grimm et al., 2017; Wang et al., 2012; She et al., 2018; She et al., 2019; Cang et al., 2014). They are activated by phosphoinositides such as PI(3,5)P_2_ and are proposed to participate in nicotinic acid adenine dinucleotide phosphate (NAADP) evoked signaling pathways (Xu and Ren, 2015; Wang et al., 2012). TPCs primarily conduct Na^+^ ions and exhibit voltage-dependent gating properties, particularly under depolarized membrane conditions, although varying degrees of Ca^2+^ permeability have been reported depending on experimental conditions and channel isoform (Li et al., 2019; She et al., 2018; Cang et al., 2014). Their activity contributes to the electrochemical gradient across the lysosomal membrane, thereby modulating vesicular trafficking dynamics (Li et al., 2019; Grimm et al., 2017; Wang et al., 2012; She et al., 2018; She et al., 2019; Cang et al., 2014). The TPC activity is strongly influenced by membrane lipid composition and bilayer physical properties, raising the possibility that membrane dynamics may contribute to mechanogating. Notably, dysregulation of TPC function has been implicated in neurodegenerative processes, including Parkinson’s disease (Li et al., 2019).

Further, TMEM175 serves as a lysosomal potassium channel that maintains membrane potential, supports luminal ion homeostasis, and preserves lysosomal function (Cang et al., 2015; Wu et al., 2023; Tang et al., 2026; Hu et al., 2022b; Wang et al., 2017). The lysosomal membrane potential, which determines the driving force for lysosomal calcium release, is strongly influenced by potassium conductance. TMEM175 stabilizes lysosomal membrane potential and supports acidification by counterbalancing vacuolar-type ATPase (V-ATPase) activity (Cang et al., 2015; Wu et al., 2023; Tang et al., 2026). Notably, TMEM175 variants have been genetically linked to Parkinson’s disease (Hu et al., 2022b), where impaired lysosomal ion homeostasis and reduced degradative capacity contribute to α-synuclein aggregation (Hu et al., 2022b).

In addition, large-conductance calcium-activated potassium (BK) channels provide feedback coupling between luminal calcium release and membrane repolarization and may contribute to the integration of ionic and membrane-associated signaling processes (Tang et al., 2026; Wang et al., 2017; Cao et al., 2015). Furthermore, several two-pore-domain potassium (K2P) family channels are sensitive to mechanical cues (Brohawn et al., 2014; Honore, 2007; Patel and Honore, 2001), particularly TWIK2, and have been proposed to contribute to lysosomal potassium conductance and ionic homeostasis (Khanra et al., 2025; Bobak et al., 2017; Huang et al., 2023; Wu et al., 2022), although their precise physiological roles in lysosomes remain incompletely understood.

Although these lysosomal channels have not been shown to function as primary inherent mechanosensors, they likely influence downstream responses to mechanically induced ionic fluxes and participate in the integration of mechanical, ionic, and metabolic signaling pathways within lysosomes. Emerging evidence further links lysosomal ion channel signaling to autophagy and cellular stress adaptation. Lysosomal channels regulate autophagosome maturation, lysosomal fusion events, nutrient sensing, and cellular stress-responsive signaling cascades that determine cellular survival (Kondratskyi et al., 2018; Brandenstein et al., 2016). Mechanical activation of lysosomal channels may therefore provide a mechanism through which cells monitor organelle integrity and adapt to cellular stress, osmotic shock, mechanical deformation, migration, and tissue remodeling.

Consistent with this concept, chloride and anion fluxes are essential for the osmotic and biophysical stability of lysosomes. LRRC8/VRAC channels (Hu et al., 2022a; Li et al., 2020), and potentially cystic fibrosis transmembrane conductance regulator (CFTR) mediated chloride signaling (Kondratskyi et al., 2018), have been implicated in the regulation of luminal chloride concentration and lysosomal volume adaptation. In particular, LRRC8/VRAC channels are well-established mediators of cell volume regulation, activated by changes in membrane dynamics and ionic strength, suggesting a similar role in lysosomal osmotic homeostasis. These channels may act as key mediators of osmotic mechanical coupling, where ion flux adjusts luminal osmolarity to counteract mechanical stress and maintain organelle integrity. By modulating luminal osmolarity, these channels contribute to cellular osmoregulation and enhance cellular survival during multiple forms of physiological stress (Hu et al., 2022a; Li et al., 2020).

Together, these observations support the existence of specialized lysosomal signaling mechanisms that are capable of integrating both osmotic and mechanical information. The physiological relevance of lysosomal mechanosignaling is increasingly apparent in human diseases. Mutations affecting lysosomal ion channels cause a broad spectrum of disorders, including lysosomal storage diseases, neurodegenerative syndromes, and inherited neurological conditions (Lloyd-Evans and Waller-Evans, 2020; Cang et al., 2015; Wu et al., 2023; Tang et al., 2026; Hu et al., 2022b; Kousi et al., 2009; Platt et al., 2018). Similarly, dysfunction of TMEM63 family members has been linked to hypomyelinating leukodystrophy (Halford et al., 2025; Yoneno et al., 2024; Yan et al., 2019; Tonduti et al., 2021; Fukumura et al., 2022), developmental and epileptic encephalopathy (Sasaki et al., 2024; Vetro et al., 2023), hereditary spastic paraplegia (Tábara et al., 2022), hearing impairment (Du et al., 2020), kidney disease (Orphal et al., 2020; Eisenreich et al., 2020; Schulz et al., 2019) and cancer progression (Zhang T. M. et al., 2023) (Figure 2; Table 2). The discovery of lysosomal mechanosensation by TMEM63A raises the intriguing possibility that defective intracellular force sensing contributes directly to disease pathogenesis and represents a previously unrecognized component of TMEM63-associated channelopathies.

Current evidence collectively supports a model in which lysosomes function as mechanosensitive intracellular organelles that can convert membrane tension into ionic and biochemical signals. The coordinated activities of lysosomal TMEM63, TRPML, TPC, TMEM175, BK, and LRRC8/VRAC channels, as well as other membrane adaptation-regulated ion signaling pathways, allow mechanical information to be integrated into metabolic, osmotic, and stress-responsive signaling pathways.

Although the molecular mechanisms of organellar mechanosignaling remain incompletely understood, recent findings establish lysosomes as important mechanosignaling centers and position the TMEM63A channel as a potential mediator linking membrane mechanics to intracellular ion homeostasis. However, future investigations of lysosomal mechanosensation will likely reveal new principles and models of intracellular force sensing and might reveal novel therapeutic opportunities for neurological, metabolic, renal, and lysosome-associated diseases.

## 5 Physiological and pathophysiological roles of TMEM63 channels

The identification of TMEM63/OSCA proteins as mechanosensitive ion channels and in mechanically regulated lipid scramblases has substantially expanded their biological relevance. TMEM63A, TMEM63B, and TMEM63C are now implicated in diverse physiological processes, ranging from nervous system development, osmosensation, pulmonary surfactant secretion, pancreatic and intestinal homeostasis, and renal stress responses to intracellular membrane organization. In parallel, human genetic studies and disease models have linked TMEM63 dysfunction to neurodevelopmental channelopathies, hereditary spastic paraplegia, sensory impairment, kidney disease, chronic pain, and cancer (Figure 2; Table 2). In the following overview, physiological functions and implications for their involvement in diseases are summarized.

### 5.1 Nervous system development, myelination, and neurodevelopmental disease

Among the three mammalian TMEM63 paralogs, TMEM63A has emerged as a particularly important regulator of central nervous system development and myelin formation (Wang Y. Y. et al., 2025; Halford et al., 2025; Dereddi et al., 2026). TMEM63A is expressed in oligodendrocyte lineage cells and promotes oligodendrocyte differentiation and maturation (Wang Y. Y. et al., 2025). Loss of TMEM63A impairs the transition of oligodendrocyte precursor cells into myelinating oligodendrocytes, whereas channel activity facilitates myelin formation (Wang Y. Y. et al., 2025). More recent studies identified TMEM63A as an evolutionarily conserved regulator of myelination across vertebrate species, supporting a fundamental role for mechanosensitive signaling in myelin development (Halford et al., 2025).

High-resolution imaging and electron microscopy revealed that TMEM63A is distributed throughout the complex architecture of oligodendrocytes, including the plasma membrane of oligodendrocyte processes, nascent myelin sheaths, and intracellular endolysosomal compartments (Halford et al., 2025). This localization pattern suggests that TMEM63A may contribute not only to plasma membrane mechanosensation but also to intracellular trafficking and organellar homeostasis during oligodendrocyte maturation and myelin development (Halford et al., 2025). TMEM63A was further shown to cycle between the plasma membrane and lysosomes, consistent with combined surface mechanosensory and intracellular trafficking roles (Halford et al., 2025).

Mechanistically, TMEM63A mediates stretch-evoked calcium influx in oligodendrocytes, enabling these cells to respond to the mechanical forces generated during axon wrapping and myelin sheath expansion (Dereddi et al., 2026). TMEM63A localizes in high-density clusters in the plasma membrane as well as in intracellular compartments (Dereddi et al., 2026). Because myelin formation requires extensive membrane expansion and remodeling, TMEM63A likely links membrane tension to cytoskeletal and signaling pathways that control myelin sheath growth and geometry (Dereddi et al., 2026). Experimental studies in zebrafish and mouse models further demonstrated that loss of TMEM63A impairs myelin sheath growth, alters sheath geometry, reduces conduction velocity, and causes locomotor deficits (Halford et al., 2025; Dereddi et al., 2026). At the molecular level, TMEM63A-dependent calcium entry regulates MYO5A-mediated transport of myelin basic protein (MBP) mRNA into developing myelin sheaths, directly linking mechanical stimuli to myelin biogenesis (Dereddi et al., 2026).

The physiological role of TMEM63A in myelination is reflected by its association with hypomyelinating leukodystrophy 19 (HLD19). Independent genetic and clinical studies identified heterozygous missense variants in TMEM63A, including *de novo* and familial cases, in patients with transient infantile hypomyelination, developmental delay, nystagmus, hypotonia, delayed motor development, and hypomyelinating leukodystrophy (Wang Y. Y. et al., 2025; Yoneno et al., 2024; Yan et al., 2019; Tonduti et al., 2021; Fukumura et al., 2022). Reported disease-associated variants include G168E, I462N, G553V, G553D, Y559H, G567S, and A632T (Wang Y. Y. et al., 2025; Yoneno et al., 2024; Yan et al., 2019; Tonduti et al., 2021; Fukumura et al., 2022). Many of these variants affect highly conserved transmembrane or pore-associated regions and have subsequently been analyzed in functional studies (Wang Y. Y. et al., 2025; Halford et al., 2025; Zheng et al., 2025; Yan et al., 2019). Functional analyses, including electrophysiological recordings, calcium imaging, and trafficking assays, indicate that several pathogenic variants reduce mechanically or osmotically evoked TMEM63A activity or impair membrane localization, consistent with a loss-of-function mechanism (Wang Y. Y. et al., 2025; Halford et al., 2025; Yan et al., 2019). Consequently, reduced stretch- or osmotic stimulus-induced calcium signaling disrupts mechanosignaling pathways required for oligodendrocyte maturation and myelin development (Wang Y. Y. et al., 2025; Halford et al., 2025; Dereddi et al., 2026). Interestingly, affected individuals frequently show substantial recovery of white matter abnormalities during childhood and adolescence, suggesting that TMEM63A dysfunction delays, rather than completely prevents, myelination (Yoneno et al., 2024; Yan et al., 2019; Tonduti et al., 2021; Fukumura et al., 2022). Some pathogenic variants also exhibit defective trafficking and reduced membrane localization (Halford et al., 2025), whereas rare variants such as S538L may be associated with altered channel regulation rather than simple loss of function (Halford et al., 2025; Zheng et al., 2025). These findings indicate that both reduced mechanosensitive signaling and aberrant channel regulation may contribute to TMEM63A-associated myelin disease.

TMEM63-dependent mechanosensation also contributes to neuronal migration. Membrane tension-dependent signaling through TMEM63B triggers calcium influx and subsequent actomyosin activation, thereby supporting soma translocation during neuronal migration (Minegishi et al., 2025). Increased plasma membrane tension generated by traction forces activates TMEM63B at the cell rear, leading to calcium influx and actomyosin contraction (Minegishi et al., 2025). Downregulation of TMEM63B abolishes traction force-induced calcium transients and disrupts coordination between leading process extension and somal movement, indicating that TMEM63B-dependent mechanosensation is required for efficient neuronal migration (Minegishi et al., 2025).

In addition to these developmental roles, TMEM63B mutations have recently emerged as a cause of developmental and epileptic encephalopathy (DEE) (Sasaki et al., 2024; Vetro et al., 2023). In contrast to TMEM63A-associated HLD19, which is mainly linked to loss-of-function variants, TMEM63B-associated disease appears to result predominantly from gain-of-function mechanisms (Vetro et al., 2023; Miyata et al., 2025). Multiple pathogenic *de novo* variants have been identified, including V44M, R433H, D459E, V463I, T481N, G580C, G580S, R660T, F697L, and an in-frame deletion of I475 (Sasaki et al., 2024; Vetro et al., 2023). Affected individuals present with developmental delay, epilepsy, intellectual disability, hypotonia, movement abnormalities, cerebral atrophy, behavioral dysfunction, and hematological abnormalities (Sasaki et al., 2024; Vetro et al., 2023).

Recent structural and functional studies revealed that several DEE-associated variants do not merely enhance ion channel activity but destabilize the closed conformation of TMEM63B and promote constitutive phospholipid scrambling (Vetro et al., 2023; Miyata et al., 2025; Lin et al., 2026). The recurrent V44M mutation disrupts plasma membrane lipid asymmetry, causing abnormal phosphatidylserine exposure and altered membrane organization (Vetro et al., 2023; Miyata et al., 2025). Structural analyses suggest that these mutations facilitate opening of a lateral lipid translocation pathway, effectively converting TMEM63 proteins into constitutively active lipid scramblases (Miyata et al., 2025; Lin et al., 2026). A homologous TMEM63A-V53M variant associated with DEE similarly promotes lipid translocation across the bilayer rather than primarily enhancing mechanically induced ion currents (Zheng et al., 2025). Mechanistically, DEE-associated gain-of-function mutations have been proposed to disrupt a hydrophobic latch near the membrane-facing surface of the protein, destabilizing the closed state and favoring conformational rearrangements that open a lateral cleft for lipid movement (Zheng et al., 2025). Whether this cleft also opens during physiological mechanical activation of wild-type TMEM63A or TMEM63B remains unresolved. Nevertheless, these findings indicate that neurodevelopmental disease can arise not only from defective ion conduction but also from disturbed membrane lipid organization and phospholipid homeostasis (Zheng et al., 2025; Vetro et al., 2023; Miyata et al., 2025; Lin et al., 2026).

### 5.2 Sensory systems, osmosensation, and pain

Mechanosensation and osmosensation represent core physiological functions of TMEM63 channels. TMEM63B is highly expressed in inner hair cells and essential for normal auditory function (Du et al., 2020). Genetic deletion causes necroptosis of outer hair cells in the cochlea and progressive hearing impairment (Du et al., 2020; Ye et al., 2024). Subsequent studies further demonstrated a role for TMEM63B in postnatal development and maintenance of cochlear sensory epithelia (Ye et al., 2024). These findings establish TMEM63B as an important component of auditory physiology and suggest that impaired TMEM63B-dependent mechanosensation can contribute to hearing dysfunction (Du et al., 2020; Ye et al., 2024).

A major advance in understanding TMEM63B physiology came from studies of interoceptive neurons in the subfornical organ, where TMEM63B functions as a mammalian osmosensor involved in thirst regulation and systemic fluid homeostasis (Yang et al., 2024; Zou et al., 2025). Hyperosmotic stimuli induce cell shrinkage and membrane deformation, leading to activation of TMEM63B in specialized interoceptive neurons (Yang et al., 2024). Genetic disruption of TMEM63B impairs neuronal responses to osmotic stress and reduces water-seeking and thirst behavior following dehydration (Yang et al., 2024). These findings were subsequently confirmed and extended by studies showing that TMEM63B is an essential component of neuronal circuits that regulate thirst, hemodynamics, and systemic water balance (Zou et al., 2025). Thus, TMEM63B links changes in extracellular osmolarity and membrane tension to neuronal signaling pathways that control interoceptive behavior and organismal homeostasis.

TMEM63 channels have also been implicated in pain signaling. TMEM63A expression is increased in non-peptidergic nociceptors in the dorsal root ganglion of patients with chronic post-amputation pain, where it may contribute to mechanical pain and modulation of chronic pain sensation (Pu et al., 2023). This increase occurs together with macrophage infiltration and pro-inflammatory cytokine release in dorsal root ganglion neurons and is supported by findings in the tibial nerve transfer mouse model, which mimics aspects of post-amputation pain (Pu et al., 2023). Although the precise causal contribution of TMEM63A to chronic pain remains to be fully defined, these observations suggest that TMEM63-dependent mechanosensation may participate in pathological sensory sensitization.

### 5.3 Lung mechanobiology and surfactant-related pulmonary dysfunction

The lung is continuously exposed to mechanical forces generated by breathing, alveolar expansion, and tissue recoil. Accordingly, pulmonary epithelial cells rely on mechanosensitive signaling pathways to adapt secretory and barrier functions to changing mechanical demands. TMEM63A and TMEM63B have been identified as critical mediators of stretch-induced responses in alveolar type II epithelial cells (Chen et al., 2024). Both channels localize to lamellar bodies, which are lysosome-related organelles specialized for surfactant storage and secretion (Chen et al., 2024). During lung inflation, TMEM63A and TMEM63B facilitate surfactant release from lamellar bodies, providing a direct link between mechanical stretch, organelle-associated mechanosensation, and pulmonary secretory function (Chen et al., 2024; Hook, 2024).

The pathophysiological importance of this mechanism is illustrated by genetic deletion studies. Loss of both TMEM63A and TMEM63B impairs inflation-induced surfactant release, leading to alveolar collapse, respiratory failure, and early lethality in mice (Chen et al., 2024). In particular, alveolar type II cell-specific deletion prevents proper lung inflation at birth (Chen et al., 2024). Because surfactant reduces alveolar surface tension and is essential for efficient gas exchange (Hallman et al., 2001), defective TMEM63-dependent surfactant secretion may contribute to pulmonary disorders characterized by abnormal mechanical stress, impaired surfactant release, or failed respiratory adaptation (Chen et al., 2024; Hook, 2024; Hallman et al., 2001). Thus, TMEM63 channels provide a molecular framework linking respiratory mechanics to secretion from lysosome-related organelles in the lung.

### 5.4 Endocrine, metabolic, and gastrointestinal homeostasis

TMEM63 channels also contribute to endocrine and metabolic homeostasis by coupling osmotic and mechanical stimuli to calcium signaling, secretion, and tissue function. Tsvilovskyy and colleagues establish TMEM63A as a key regulator of intracellular calcium homeostasis within pancreatic acinar cells, where TMEM63A regulates calcium release by TPCs in acidic stores (Tsvilovskyy et al., 2024). For better understanding, the authors renamed TMEM63A as organellar calcium regulator of organellar calcium release channels (OCaR1) (Tsvilovskyy et al., 2024). It was shown that OCaR1 is primarily present in endolysosomes and secretory granules of acinar cells, where it protects acinar cells from uncontrolled exocytosis of secretory vesicles with pancreatic enzymes, which affects pancreatic health (Tsvilovskyy et al., 2024; Patel and Yule, 2024). Functionally, OCaR1 restrains calcium spikes in these acidic organelles by dampening TPC1/2 channel activity and limiting nicotinic acid adenine dinucleotide phosphate-evoked calcium signals (Tsvilovskyy et al., 2024). In this way, OCaR1 protects acinar cells from uncontrolled exocytosis of digestive enzyme-containing secretory vesicles (Tsvilovskyy et al., 2024; Patel and Yule, 2024).

Loss of OCaR1 disrupts this autoregulatory control, leading to spontaneous calcium oscillations and exaggerated calcium responses to stimulation with cholecystokinin (CCK) (Tsvilovskyy et al., 2024). This dysregulation promotes excessive exocytosis of digestive enzymes and predisposes acinar cells to injury. Consistent with this cellular phenotype, OCaR1-deficient mice show increased susceptibility to both acute and chronic pancreatitis (Tsvilovskyy et al., 2024). These findings position TMEM63A/OCaR1 as an intrinsic gatekeeper of organellar calcium signaling and secretory granule homeostasis in pancreatic acinar cells.

In pancreatic β-cells, TMEM63B functions as a stretch-activated ion channel that contributes to glucose-stimulated insulin secretion (Tu et al., 2025). Glucose metabolism induces β-cell swelling, which activates TMEM63B and promotes membrane depolarization, calcium influx, and insulin granule exocytosis (Tu et al., 2025). Loss of TMEM63B impairs glucose-stimulated insulin secretion and reduces β-cell responsiveness, indicating that mechanosensitive signaling participates in the regulation of glucose metabolism (Tu et al., 2025). Because metabolic activity is accompanied by changes in cell volume, osmolarity, and membrane tension, TMEM63B may act as a sensor that converts these physical changes into calcium-dependent endocrine output.

Beyond the pancreas, TMEM63B has been implicated in gastrointestinal physiology. TMEM63B is highly expressed in intestinal stem cells and regulates intestinal motility as well as the proliferation of intestinal stem cells (Tu et al., 2024). Conditional deletion of TMEM63B in intestinal stem cells reduces intestinal motility, impairs digestion, and decreases stem-cell proliferation (Tu et al., 2024). Given that intestinal tissues experience continuous mechanical and osmotic challenges associated with luminal flow, nutrient absorption, and epithelial renewal, TMEM63-dependent mechanosensation may coordinate digestive function, epithelial homeostasis, and tissue regeneration (Tu et al., 2024).

### 5.5 Kidney function, podocyte stress response, and renal disease

The kidney is exposed to substantial mechanical stress generated from glomerular filtration, tubular fluid flow, osmotic gradients and blood pressure fluctuations. Several studies have implicated TMEM63 family members, especially TMEM63C, in renal physiology and adaptation to mechanical and hormonal stress (Orphal et al., 2020; Eisenreich et al., 2020; Schulz et al., 2019). Genetic analyses of hypertensive rat models identified Tmem63c as a candidate susceptibility gene associated with kidney damage (Schulz et al., 2019). In renal cells, TMEM63C expression is regulated by transforming growth factor-β (TGF-β) signaling and microRNA (miRNA-564)-mediated pathways (Orphal et al., 2020).

In podocytes, TMEM63C appears to function as a pro-survival factor in angiotensin II-treated podocytes, suggesting a protective role during hemodynamic and mechanical stress (Eisenreich et al., 2020). Podocytes are continuously subjected to mechanical forces generated by the glomerular filtration pressure. Eisenreich and colleagues investigated the regulation and function of TMEM63C in the development of pathogenic chronic kidney disease (Eisenreich et al., 2020). Angiotensin II, a potent vasoconstrictor in the body (Harrison-Bernard, 2009), induces TMEM63C expression in podocytes via the NF-κB signaling pathway (Eisenreich et al., 2020) suggesting that TMEM63C participates in adaptive responses to hemodynamic and mechanical stress (Eisenreich et al., 2020). Although the precise molecular function of TMEM63C remains incompletely understood, these findings indicate that TMEM63C may contribute to preserving podocyte integrity under pathological stress conditions (Eisenreich et al., 2020).

Renal disease associations further support a pathophysiological role for TMEM63C. Reduced TMEM63C expression has been associated with albuminuria, focal segmental glomerulosclerosis, and chronic kidney disease, although direct causal relationships remain to be fully established (Orphal et al., 2020; Eisenreich et al., 2020; Schulz et al., 2019). Importantly, evidence for mechanosensitive ion channel activity of TMEM63C in kidney cells is still limited. Thus, TMEM63C should currently be viewed as a candidate regulator of podocyte stress adaptation and renal disease progression rather than as a fully established renal mechanosensitive channel.

### 5.6 Intracellular membrane organization and hereditary spastic paraplegia

TMEM63C has also been linked to autosomal recessive hereditary spastic paraplegia, expanding the disease spectrum of the TMEM63 family beyond TMEM63A- and TMEM63B-associated neurodevelopmental disorders (Tábara et al., 2022). Biallelic pathogenic variants, including N547K, W406G, T195* (a premature stop codon that terminates protein synthesis), and Y525L, have been identified in affected individuals with progressive lower-limb spasticity, gait abnormalities, motor dysfunction, and mild intellectual disability (Tábara et al., 2022).

Unlike TMEM63A and TMEM63B, which have been studied primarily in the context of plasma membrane or lysosome-related mechanosensation, TMEM63C appears to function predominantly within intracellular membrane systems. TMEM63C localizes to the endoplasmic reticulum and is enriched at ER-mitochondria contact sites, where it contributes to maintenance of organelle morphology and phospholipid homeostasis (Tábara et al., 2022). Silencing of TMEM63C disrupts ER morphology, mitochondrial architecture, and inter-organelle contacts, suggesting that defective ER-mitochondria communication contributes to disease pathogenesis (Tábara et al., 2022). TMEM63C deficiency also alters phospholipid homeostasis and membrane organization, supporting a role in intracellular membrane regulation (Tábara et al., 2022). Current evidence therefore suggests that TMEM63C-associated hereditary spastic paraplegia may result primarily from perturbation of intracellular membrane dynamics rather than from defective plasma membrane mechanosensation alone (Tábara et al., 2022).

### 5.7 Cell migration, tissue remodeling, and cancer biology

Mechanical forces are fundamental regulators of cell migration, tissue remodeling and cancer progression. Several studies suggest that TMEM63 proteins participate in these processes by coupling membrane deformation to intracellular signaling pathways. Overexpression of TMEM63B enhances migratory behavior in cultured cells, supporting a role in cellular responses to osmotic and mechanical stress (Marques et al., 2019).

As mentioned before, TMEM63B plays a role in neuronal migration (Minegishi et al., 2025). An increase in plasma membrane tension due to traction forces activates TMEM63B channels, which causes calcium influx and actomyosin contraction at the cell rear, resulting in soma translocation (Minegishi et al., 2025). Downregulation of TMEM63B abolished traction force-induced calcium transients and impaired the interplay between the leading process extension and the somal movement in neurons (Minegishi et al., 2025). These findings suggest that TMEM63B-dependent mechanosensation is important for efficient neuronal migration. These findings indicate that TMEM63-dependent mechanosensation may contribute more broadly to developmental remodeling and tissue dynamics.

Emerging evidence also links TMEM63 proteins to cancer biology. A study by Zhang et al. describes a novel, autophagy-based degradation pathway mediated by the selective autophagy receptor toll-interacting protein (TOLLIP) (Zhang T. M. et al., 2023). This pathway connects valosin-containing protein (VCP), TMEM63A, and degradation in endoplasmic reticulum protein 1 (DERL1), and the study suggests that TMEM63A functions as an oncogene promoting triple-negative breast cancer progression through regulation of autophagic pathways and cellular stress responses (Zhang T. M. et al., 2023). Elevated TMEM63A expression has been associated with proliferation, migration, invasion, and metastasis formation in triple-negative breast cancer models (Zhang T. M. et al., 2023). Although the mechanistic relationship between TMEM63A channel activity, lipid remodeling, and tumor progression remains to be fully established, these findings suggest that TMEM63-dependent mechanosignaling may influence cancer cell adaptation to mechanically heterogeneous tumor microenvironments and could represent a potential therapeutic target (Zhang T. M. et al., 2023).

Taken together, TMEM63 proteins participate in a broad range of physiological systems, and their dysfunction extends from monogenic channelopathies to complex organ-specific diseases. The currently recognized disease spectrum is dominated by neurodevelopmental disorders, including TMEM63A-associated HLD19 and TMEM63B-associated DEE, but accumulating evidence implicates TMEM63 channels in sensory dysfunction, chronic pain, pulmonary adaptation, pancreatitis, metabolic regulation, gastrointestinal homeostasis, renal disease, hereditary spastic paraplegia, and cancer. A key emerging concept is that TMEM63-associated pathophysiology may reflect disruption of both ion conduction and membrane lipid organization. Future studies integrating structural biology, mechanobiology, human genetics, and organelle physiology will be essential to determine how TMEM63-dependent mechanosensation and lipid remodeling contribute to disease mechanisms and whether these pathways can be therapeutically targeted.

## 6 Pharmacological and therapeutic perspectives

Despite the rapidly growing understanding of TMEM63/OSCA channel biology, pharmacological targeting of TMEM63 proteins remains at an early stage. To date, selective small-molecule activators or inhibitors that can be used as routine pharmacological tools for mammalian TMEM63A, TMEM63B or TMEM63C have not yet been established. This lack of selective modulators currently limits direct experimental dissection of TMEM63 channel function and prevents immediate therapeutic translation. Nevertheless, recent structural, electrophysiological, and disease-genetic studies provide an increasingly strong framework for future drug discovery (Zheng et al., 2023; Zhang M. et al., 2023; Qin et al., 2023; Han et al., 2024; Shan et al., 2024; Zheng et al., 2025; Vetro et al., 2023; Miyata et al., 2025; Lin et al., 2026; Wu et al., 2024).

Several conceptually distinct therapeutic strategies can be envisioned. For loss-of-function TMEM63A variants associated with hypomyelinating leukodystrophy, therapeutic approaches would need to restore channel function, membrane trafficking, or expression in oligodendrocytes and possibly endolysosomal compartments (Wang Y. Y. et al., 2025; Halford et al., 2025; Dereddi et al., 2026; Yoneno et al., 2024; Yan et al., 2019; Tonduti et al., 2021; Fukumura et al., 2022). Potential future strategies may include gene replacement, mRNA-based approaches, or small molecules that improve folding, trafficking, or channel activation. In contrast, gain-of-function TMEM63B variants associated with developmental and epileptic encephalopathy may require approaches that reduce excessive channel activity or constitutive phospholipid scrambling (Sasaki et al., 2024; Vetro et al., 2023; Miyata et al., 2025; Lin et al., 2026). In this context, small molecules stabilizing the closed conformation, inhibitors of aberrant lipid scrambling, or allele-selective gene-silencing strategies could represent future therapeutic concepts.

The emerging lysosomal function of TMEM63A opens an additional pharmacological perspective. Enhancing TMEM63A-dependent lysosomal mechanoresilience might be beneficial under conditions in which lysosomes experience acute osmotic or mechanical stress, whereas excessive activity could disturb organellar ion and lysosomal volume homeostasis. Thus, any future intervention will require careful attention to subcellular localization, tissue specificity, and disease context. This is particularly important because TMEM63 channels contribute to multiple physiological systems, including myelination, pulmonary surfactant secretion, pancreatic and intestinal homeostasis, renal stress responses, and sensory signaling. Comparisons with other lysosomal ion channels, such as TRPML1, TPCs, and TMEM175, illustrate that lysosomal channels can be pharmacologically tractable, but they also highlight the need for selective and compartment-aware modulators (Kondratskyi et al., 2025; Li et al., 2019; Cang et al., 2014; Cang et al., 2015; Wu et al., 2023; Tang et al., 2026).

Overall, TMEM63 proteins should currently be viewed as promising but still largely unexplored pharmacological targets. Future development of isoform-selective modulators, structure-guided screening approaches, and disease-relevant cellular models will be essential to determine whether TMEM63-dependent mechanosensation and lipid scrambling can be therapeutically exploited.

## 7 Conclusions and outlook

The TMEM63/OSCA family has emerged as a fundamentally important class of mechanosensitive membrane proteins that are evolutionarily conserved across eukaryotes (Figure 1). Originally identified as osmosensitive channels in plants, TMEM63 proteins are now recognized as versatile mechanosensors that directly convert membrane deformation into cellular signals through mechanisms closely linked to lipid-protein interactions and bilayer mechanics (Murthy et al., 2018; Hou et al., 2014; Yuan et al., 2014). Structural studies have revealed a unique channel architecture that enables force sensing through membrane-associated gating mechanisms, establishing TMEM63 proteins as representative examples of the *force-from-lipids* principle (Zheng et al., 2023; Zhang M. et al., 2023; Qin et al., 2023; Han et al., 2024; Shan et al., 2024; Teng et al., 2015; Anishkin et al., 2014; Pliotas et al., 2015; Bavi et al., 2023; Martinac et al., 1990; Perozo et al., 2002; Wu et al., 2024). Recent advances have substantially expanded the functional repertoire of this channel family. Beyond their established role as mechanically activated ion channels, TMEM63 proteins have been identified as mechanically regulated phospholipid scramblases capable of remodeling membrane lipid asymmetry in response to alterations in membrane architecture and mechanical stress (Zheng et al., 2025; Vetro et al., 2023; Miyata et al., 2025; Lin et al., 2026; Freichel et al., 2024). This dual functionality places TMEM63 proteins at the intersection of ion homeostasis, membrane organization and mechanobiology, supporting the emerging view that these proteins function as multifunctional membrane stress sensors rather than merely as canonical ion channels.

The physiological importance of TMEM63 channels is increasingly evident across diverse tissues and organ systems (Figure 2). TMEM63A has emerged as a critical regulator of oligodendrocyte differentiation and developmental myelination (Wang Y. Y. et al., 2025; Halford et al., 2025; Dereddi et al., 2026), whereas TMEM63B functions as an osmosensor controlling hearing (Du et al., 2020; Ye et al., 2024), thirst perception (Yang et al., 2024; Zou et al., 2025), endocrine signaling (Tu et al., 2025), and intestinal physiology (Tu et al., 2024). TMEM63C contributes to intracellular membrane homeostasis and appears to play important roles in renal function (Orphal et al., 2020; Eisenreich et al., 2020; Schulz et al., 2019) and neuronal morphogenesis (Tábara et al., 2022). Furthermore, the recent discovery of TMEM63A as a mechanosensitive ion channel residing in endolysosomes and the endoplasmic reticulum has broadened the scope of mechanosensation beyond the plasma membrane and underscores the emerging role of intracellular organelles as force-sensing hubs (Tsvilovskyy et al., 2024; Patel and Yule, 2024; Tábara et al., 2022; Li et al., 2024). Together, these findings indicate that TMEM63 proteins participate in multiple physiological processes that require adaptation to mechanical, osmotic and metabolic cellular stress.

Human genetics has provided compelling evidence for the clinical significance of TMEM63-mediated signaling. Pathogenic variants in TMEM63A, TMEM63B and TMEM63C cause distinct neurological disorders, including hypomyelinating leukodystrophy (Halford et al., 2025; Dereddi et al., 2026; Yoneno et al., 2024; Yan et al., 2019; Tonduti et al., 2021; Fukumura et al., 2022), developmental and epileptic encephalopathy (Vetro et al., 2023), and hereditary spastic paraplegia (Sasaki et al., 2024; Tábara et al., 2022). Importantly, recent mechanistic studies demonstrate that disease-associated mutations can disrupt both ion channel activity and phospholipid scrambling, revealing previously unrecognized pathogenic mechanisms involving membrane lipid dysregulation and deformation (Zheng et al., 2025; Vetro et al., 2023; Miyata et al., 2025; Lin et al., 2026).

Importantly, the identification of TMEM63A as a lysosomal mechanosensitive ion channel (Table 1) has created a direct conceptual link between mechanobiology and lysosomal ion-channel signaling (Li et al., 2024). Together with established lysosomal channels such as TRPML1, TPC1/2, and TMEM175, TMEM63A supports a model in which lysosomes function not only as degradative organelles but also as dynamic mechanosensitive signaling hubs capable of integrating mechanical, osmotic, metabolic, and stress-related information (Kondratskyi et al., 2025; Xu and Ren, 2015; Li et al., 2019; Lloyd-Evans and Waller-Evans, 2020; Hu et al., 2022a; Dong et al., 2010; Venkatachalam et al., 2015; Grimm et al., 2017; Wang et al., 2014; Cao et al., 2017; Liu and Montell, 2015; Wang et al., 2012; She et al., 2018; Cang et al., 2014; Cang et al., 2015; Wu et al., 2023; Tang et al., 2026; Huang et al., 2023; Wu et al., 2022; Jaslan et al., 2020). This concept expands current views of lysosomal physiology and suggests that intracellular force sensing may represent a previously underappreciated regulator of cellular homeostasis.

However, despite remarkable advances in recent years, many fundamental aspects of TMEM63 biology remain unresolved. One of the most important outstanding questions concerns the molecular relationship between ion-channel activity and phospholipid scrambling. Recent studies have demonstrated that TMEM63 proteins can mediate both mechanically activated ion permeation and membrane lipid translocation, yet it remains unclear whether these functions occur simultaneously within a common conformational state or represent distinct mechanisms of channel activation (Zheng et al., 2025; Vetro et al., 2023; Miyata et al., 2025; Lin et al., 2026; Freichel et al., 2024). Resolving this issue will require high-resolution structural studies capturing multiple mechanically activated conformations together with functional analyses capable of separating ion-conducting and lipid-scrambling activities. Such investigations will be critical not only for understanding TMEM63 channel physiology but also for elucidating the molecular basis of TMEM63-associated diseases.

Another major challenge is defining how mechanical forces are sensed and integrated within intracellular organelles. The recent identification of TMEM63A as a lysosomal mechanosensitive ion channel has introduced the concept of organellar mechanosensation and suggests that intracellular membranes actively monitor their physical state (Li et al., 2024). However, the nature of the mechanical stimuli encountered by lysosomes under physiological conditions remains incompletely understood. Lysosomes continuously undergo membrane remodeling during fusion and fission events, intracellular trafficking, cargo accumulation, autophagy, and osmotic adaptation (Figure 3A), all of which have the potential to alter membrane tension and curvature. Future studies will need to establish how these physical forces are generated, how they are detected by TMEM63A and possible other lysosomal channels, and how mechanical information is integrated with established metabolic and stress-responsive signaling pathways. More broadly, it remains unknown whether analogous mechanosensitive systems operate in other intracellular organelles, including endosomes, autophagosomes, mitochondria, the endoplasmic reticulum, and the Golgi apparatus.

The expanding spectrum of physiological functions associated with TMEM63 proteins also raises important questions regarding tissue-specific regulation and signaling. Although TMEM63A, TMEM63B, and TMEM63C share considerable structural similarity, accumulating evidence suggests that individual family members fulfill highly specialized functions in distinct cellular conditions. Understanding how channel activity is regulated by membrane composition, lipid environment, post-translational modification, protein-protein interactions, and mechanical cues will be essential for explaining their diverse physiological roles. In particular, the recent discovery that membrane cholesterol and lipid organization strongly influence TMEM63 activity highlights the need to better understand how membrane composition and organization shapes mechanosensitive signaling under physiological and pathological conditions (Miyata et al., 2025; Lin et al., 2026).

Human genetics has further emphasized the clinical importance of TMEM63-mediated signaling. Disease-causing variants have already been linked to hypomyelinating leukodystrophy, developmental and epileptic encephalopathy, hereditary spastic paraplegia, and additional neurological disorders (Halford et al., 2025; Dereddi et al., 2026; Yoneno et al., 2024; Yan et al., 2019; Tonduti et al., 2021; Fukumura et al., 2022; Sasaki et al., 2024; Vetro et al., 2023; Tábara et al., 2022). Nevertheless, the mechanisms by which altered mechanosensation, ion flux, lipid scrambling, or organellar signaling contribute to disease pathogenesis remain only partially understood. Future work combining patient-derived cells, organoids, advanced animal models, and genome-editing technologies will be crucial for establishing genotype-phenotype relationships and identifying disease-causing signaling pathways. Such studies may also reveal whether TMEM63 dysfunction contributes more broadly to common disorders involving impaired membrane homeostasis, lysosomal dysfunction, neurodegeneration, metabolic disease, or cancer.

From a translational perspective, TMEM63 proteins represent attractive, but largely unexplored therapeutic targets. The availability of high-resolution structural information and increasing knowledge of disease-associated variants create opportunities for structure-guided drug discovery. Potential therapeutic strategies may include pharmacological modulation of mechanosensitive gating, selective regulation of phospholipid scrambling activity, manipulation of membrane lipid imbalances, or of lysosomal mechanosignaling. Although no TMEM63-directed therapies currently exist, the rapid pace of new discoveries in the field of mechanobiology and lysosomal ion-channel research suggests that therapeutic investigation of these pathways may become feasible in the future.

In summary, TMEM63 proteins have evolved into multifunctional mechanosensitive membrane proteins that integrate ion conduction, lipid scrambling, and organelle signaling. Their study is reshaping current concepts of mechanosensation by extending force sensing beyond the plasma membrane and highlighting the central role of intracellular membranes in cellular adaptation to cellular stress. Their ability to couple membrane mechanics to ion transport, lipid remodeling, and intracellular signaling places them at the intersection of mechanobiology, membrane homeostasis, and organelle physiology. Continued integration of structural biology, mechanobiology, lysosome research, and human genetics is expected to uncover new principles of cellular force sensing and membrane regulation. As the field continues to expand, TMEM63 proteins are likely to provide important insights into how cells sense and adapt to their physical environment and may ultimately emerge as therapeutically relevant targets for diseases linked to defective mechanosensation, membrane homeostasis, and organelle function.
